# Erectile Function and Sexual Behavior: A Review of the Role of Nitric Oxide in the Central Nervous System

**DOI:** 10.3390/biom11121866

**Published:** 2021-12-11

**Authors:** Maria Rosaria Melis, Antonio Argiolas

**Affiliations:** Department of Biomedical Sciences, Section of Neurosciences and Clinical Pharmacology, University of Cagliari, 09124 Cagliari, Italy; argiolas@unica.it

**Keywords:** nitric oxide, nitric oxide donors, nitric oxide targets, penile erection, sexual behavior, rat

## Abstract

Nitric oxide (NO), the neuromodulator/neurotransmitter formed from l-arginine by neuronal, endothelial and inducible NO synthases, is involved in numerous functions across the body, from the control of arterial blood pressure to penile erection, and at central level from energy homeostasis regulation to memory, learning and sexual behavior. The aim of this work is to review earlier studies showing that NO plays a role in erectile function and sexual behavior in the hypothalamus and its paraventricular nucleus and the medial preoptic area, and integrate these findings with those of recent studies on this matter. This revisitation shows that NO influences erectile function and sexual behavior in males and females by acting not only in the paraventricular nucleus and medial preoptic area but also in extrahypothalamic brain areas, often with different mechanisms. Most importantly, since these areas are strictly interconnected with the paraventricular nucleus and medial preoptic area, send to and receive neural projections from the spinal cord, in which sexual communication between brain and genital apparatus takes place, this review reveals that central NO participates in concert with neurotransmitters/neuropeptides to a neural circuit controlling both the consummatory (penile erection, copulation, lordosis) and appetitive components (sexual motivation, arousal, reward) of sexual behavior.

## 1. Introduction

Nitric oxide (NO), the gaseous highly diffusible compound produced from the amino acid l-arginine by NO synthases, a family of Ca^2+^-calmodulin-dependent iron containing enzymes (e.g., neuronal, endothelial or inducible NO synthase) ([1,2,3,4] and references therein), has been added to the list of neurotransmitters and/or neuromodulators at the level of the peripheral and central nervous system in the 1990s [5,6,7,8,9,10,11,12]. Since then, NO has been involved in numerous functions at peripheral and central level. Among these, one of the best known is certainly its key role in relaxing cavernous corpora smooth muscles at the penile level (a key passage for the induction of penile erection) by activating guanylate cyclase, the enzyme that converts guanosine triphosphate (GTP) in cyclic guanosine monophosphate (cGMP), the second messenger that—together with the other second messenger cyclic adenosine monophosphate (cAMP) formed from adenosine triphosphate (ATP) by the enzyme adenylate cyclase—is well known for its key role in practically every cellular function in all living animals. NO in cavernous corpora is produced mainly by the endothelial NO synthase isoform localized in endothelial cells that overlay smooth muscle cells upon activation by an increased Ca^2+^ influx secondary to the activity of cholinergic and non-cholinergic neurons that impinge on them. However, at penile level also the neuronal NO synthase isoform is present, mainly in non-adrenergic–non-cholinergic neurons, which participate in the relaxation of cavernous smooth muscles together with the cholinergic ones (reviews of the mechanism controlling cavernous corpora smooth muscles relaxation are found in [9,13,14,15,16,17]). The potency of NO in activating guanylate cyclase and increasing cGMP concentration that mediates the relaxation of cavernous smooth muscles led researchers to consider NO as the key physiological neurotransmitter of penile erection at local level [10,11,18]. Together with the selective presence in cavernous corpora smooth muscles of phosphodiesterase type V enzyme, which selectively inactivates cGMP to GMP leading to the end of the cavernous corpora relaxation and then of penile erection, this has opened the way to the discovery of selective, orally active inhibitors of this phosphodiesterase enzyme isoform clinically used today for the treatment of erectile dysfunction (reviews on phosphodiesterase type V and its functions are found in [19,20]). Studies with selective endothelial or neuronal NO synthase knockout mice revealed that both NO synthase isoforms play a role in the relaxation of the cavernous smooth muscles, with the neuronal isoform playing a main role in the initiation and the endothelial one in the maintenance of penile erection [21]. Most importantly, these studies revealed also that neuronal NO synthase knockout mice mate normally and have a NO-dependent erectile response [21]. This is due to endothelial NO synthase and alternative spliced neuronal NO synthase variants surviving gene deletion, synthetizing NO and maintaining erectile function even in the absence of neuronal NO synthase [22,23].

NO synthase isoforms are also present in the central nervous system, where a NO-guanylate cyclase operating system has been also identified in several brain areas. Among these, particular attention has been dedicated to the hippocampus and the cerebellum, which play a key role in learning and memory formation and in which mechanisms strongly related to these central functions, such as long-term potentiation and long-term depression, are well known to take place [6,7,12,24,25]. In these areas, neuronal NO synthase that produces NO is localized in the cell bodies of neurons on which impinge excitatory (usually glutamatergic) synapses. The activation of these neurons through excitatory amino acid receptors of the AMPA [(±)-a-amino-3-hydroxy-5-methyl-isoxaxole-4-propionic acid] first and then of NMDA (N-methyl-D-aspartic acid) subtypes (NMDA receptors, which are coupled to voltage-dependent Ca^2+^ channels), leads to an increased Ca^2+^ influx and subsequent NO synthase activation in these neuronal cell bodies. Newly produced NO travels as a retrograde messenger from the cell bodies in which it has been produced to the excitatory glutamatergic synapses, where guanylate cyclase is localized, activating its production of cGMP which activates the mechanisms that produce long term potentiation in the hippocampus or long-term depression in the cerebellum or vice versa, depending on the neuronal system involved [6,7,12,24]. This initial picture is changing continuously, with the discovery of new modes of operation of NO ranging from local signaling to volume-type transmission across various brain regions and nuclei ([26] and references therein).

Another area of the brain rich in NO synthase is the hypothalamus and in particular the paraventricular nucleus (PVN), which appears to be one of the brain nuclei richest in NO synthase [27]. Detailed immunocytochemical studies have shown that the NO synthase in the PVN is localized in the cell bodies and dendrites of neurons, which contain oxytocin, vasopressin and somatostatin [9,28,29,30] often found close to neurons containing corticotrophin-releasing hormone (CRH), leading researchers to suggest that NO in the PVN might be involved in the release of these two hormones in the neurohypophysis and of CRH in the median eminence ([31] and references therein). NO synthase was also detected and found involved in the activation of luteinizing hormone-releasing hormone (LH-RH) neurons in the medial preoptic area ([31] and references therein). The localization of a Ca^2+^-dependent NO synthase in PVN oxytocinergic neurons [8] in close proximity with dopamine [32,33], serotonin (5-HT) [34] and excitatory amino acid projections [35] and the above-mentioned possible involvement of NO in the release of CRH that controls corticotrophin (ACTH) release, and of LH-RH that control the release of both follicle stimulating hormone (FSH) and luteinizing hormone (LH) from the adenohypophysis, led the authors of this review and other research groups to investigate on a possible role of NO in the central control of erectile function and sexual behavior first in the PVN [36,37,38,39,40,41,42,43] and in the medial preoptic area [44,45] and later in other brain areas. It is now clear that NO plays a role in the control of erectile function and sexual behavior not only at the hypothalamic and medial preoptic area level, but also in other extrahypothalamic brain areas. Among these, the most studied are the hippocampal formation [46], the ventral tegmental area [47,48], the ventral subiculum of the hippocampus, the posteromedial cortical nucleus of the amygdala [49,50,51], the ventral medulla and the spinal cord [52]. In many of these areas NO exerts its sexual effects by acting in concert with other neurotransmitters and/or neuropeptides (see Section 6 and [53,54,55,56,57]). Against the role of central NO in erectile function and sexual behavior supported by the above studies, mating and penile erection are normal in neuronal NO synthase knockout mice [21], in spite of an excess of inappropriate sexual behavior and a marked increase in aggressive behavior [58]. In fact, this finding suggests that this NO synthase isoform is not necessary for sexual behavior, apparently ruling out the involvement of central NO in erectile function and sexual behavior. It is likely that this discrepancy is only apparent, first because the other NO synthase isoforms are also present in the central nervous system and can produce NO to replace that missing for the absence of neuronal NO synthase as found at penile level (see above), and second, because this finding may simply indicate only that NO is one of the many agents controlling sexual behavior. Thus, it is likely that the failure of the absence of neuronal NO synthase to abolish/reduce mating and penile erection reflects the redundancy of systems involved in reproductive physiology, which has an evolutionary origin, as it is fundamental to guarantee the passage of genes to the next generations and thus the species survival, rather than showing no role of NO in erectile function and sexual behavior [57]. However, the hypothesis that NO-synthase knockout mice copulate normally may be due to the fact that erectile function and copulatory behavior in male mice are organized differently from those of male rats cannot be completely ruled out. In fact, (i) while male rats need a few minutes to reach the first ejaculation, which may be followed by many others, male mice need much more time to reach only one ejaculation rarely followed by others [59,60,61] and (ii) differences in the spinal control of penile reflexes have been found to occur between male mice and rats [62].

This review has been prepared by considering the results of numerous published high-quality studies, including those made in the authors’ laboratories, on the role of central NO in erectile function and sexual behavior in laboratory animals (mainly rats), from penile erection in males to copulation in males and females, and sexual intercourse in humans when available, in order to provide an updated picture of the most recent discoveries and to identify new possible advances that may be useful for realizing new strategies based on the modulation of central NO activity for the therapy of erectile dysfunctions and other sexual disorders.

## 2. Central NO and Erectile Function

Penile erection is considered the main component of the male sexual response, as it allows sexual intercourse to occur with the female counterpart. It is produced by a complex neural central and peripheral interaction that induces muscle and vascular changes at the level of the erectile tissues of the male genital apparatus (cavernous corpora, corpus spongiosum, bulbocavernosus and ischiocavernosus muscles and other perineal muscles, which are differently organized and controlled by spinal cord projections in males and females). This is further complicated by humoral and endocrine influences, exerted mainly by testosterone and its metabolites estradiol and 5α-dihydrotestosterone, which take place at central and peripheral levels. Although the aim of this review is to focus on the sexual effects of central NO, it is important to recall that testosterone and its metabolites are key regulators of male erectile function and sexual behavior since, through the modulation of genomic processes, they exert upstream effects on all signaling pathways activated by all the neurotransmitters and neuropeptides cited in this review, including NO synthase itself at both central and peripheral sites [16,59,60].

**Figure 1:**
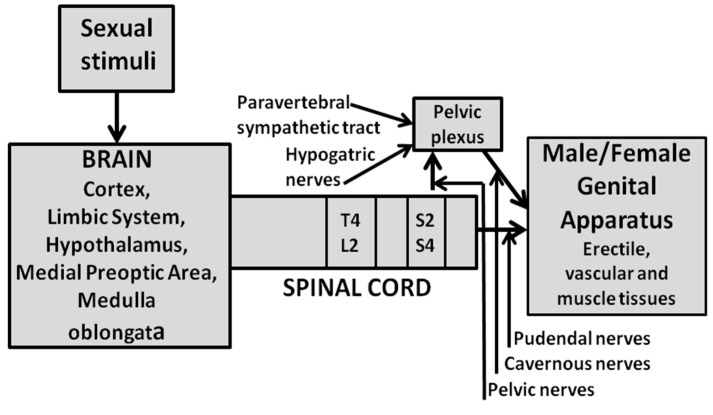
A schematic representation of the neural pathways controlling the male and female genital apparatus. When sexual (visual, auditory, olfactory, tactile and even imaginative in humans) stimuli reach the brain high centers, this activates neural pathways, to date still unknown, leading to penile erection in males and clitoris erection and vaginal lubrication in females, allowing sexual intercourse. These pathways travel from the brain, mainly from the hypothalamus and its nuclei (paraventricular nucleus, ventromedial nucleus), and medial preoptic area, through the medulla oblongata and the spinal cord, to the genital apparatus. The latter is innervated by pudendal nerves originating from the sacral S2–S4 spinal tract and containing the primary afferent sensory from and motor pathways to the penis in the male and to the clitoris in the female, and by cavernous nerves containing the primary efferent sympathetic and parasympathetic pathways originating in the pelvic plexuses. These receive neural inputs from (i) pelvic nerves originating in the sacral S2–S4 spinal tract, (ii) hypogastric nerves originating in the thoracic-lumbar (T4-L2) spinal tract and (iii) post-ganglia fibers originating from the paravertebral sympathetic ganglia of the thoracic–lumbar tract of the spinal cord (T11-L2). Details are found in [15,16,17,40,53,54,56,57,59,60,63,64,65,67,68,75].

Penile erection may occur not only during sexual activity but also in other contexts, such as after simple manipulation of the genitalia, or during sleep or erotic fantasies in humans, or in male rats put in the presence of an inaccessible receptive female (non-contact erections), or after treatment with several classes of drugs (i.e., dopamine agonists, serotonin agonists, NO donors, phosphodiesterase inhibitors, soluble guanylate cyclase activators, RhoA-Rho kinase inhibitors, etc.) and neuropeptides [i.e., adrenocorticotropin (ACTH)-melanocyte stimulating hormone (α-MSH)-related peptides, oxytocin, hexarelin analogues, VGF-related peptides (produced by proteolytic cleavage of the protein VGF) and others], acting in the central nervous system or peripherally [16,40,54,55,56,57,63,64,65,66,67,68,69,70,71,72,73,74,75]. Depending on the context in which penile erection occurs, it is generally accepted that different central and peripheral neural and/or humoral endocrine mechanisms may participate in the regulation of this sexual response, often in a very complex manner [67] (see Figure 1 for a schematic representation of central and peripheral neural pathways controlling erectile function and sexual behavior in mammals). The involvement of central NO in the control of penile erection was first shown in the 1990s by studies that revealed the inhibitory effect of NO synthase inhibitors given not only systemically (i.e., by acting mainly at the penile level) but also into the lateral ventricles (i.c.v.) (i.e., by acting at the central level) on this sexual response induced by several drugs and peptides (often together with yawning) in male rats [37,40,76]. These studies were followed by other studies showing that these compounds were also able to reduce the expression of penile erection that takes place in physiological contexts, for example when sexually potent male rats are put into the presence of an inaccessible sexually receptive female rats (non-contact erections) or when copulation was allowed (e.g., when in copula penile erections take place) [77], and even when penile erections are induced by direct stimulation of the penis (e.g., reflex erections) [42].

### 2.1. Central NO Facilitates Erectile Function in Male Rats: Effect of NO Synthase Inhibitors

As recalled above, in the 1990s the involvement of central NO in the control of penile erection was first shown by studies that revealed an inhibitory effect of NO synthase inhibitors given endovenously or i.c.v. on these responses induced by drugs (i.e., apomorphine, a mixed D1/D2 dopamine receptor agonist given systemically or into the PVN and the excitatory amino acid NMDA, agonist of the NMDA receptor, but not AMPA, agonist of the AMPA receptor or trans (±)-1-amino-1,3-cyclopentane-dicarboxilic acid (ACPD), agonist of the metabotropic receptor [78], given into the PVN or 5-HT receptor agonists of the 5-HT_1C_ subtype [i.e., 1-(3-chlorophenyl)-piperazine (m-CPP) and N-(3-trifluoro-methylphenyl)-piperazine (TFMPP)] given systemically, and peptides (i.e., oxytocin given i.c.v. or into the PVN and ACTH 1-24 given i.c.v.) in male rats (Table 1) [36,37,41,77,79,80,81,82,83,84]. In these earlier studies, two well-characterized competitive inhibitors, namely l-arginine analogs, such as N^G^-nitro-l-arginine methyl ester (L-NAME), N^G^-monomethyl-l-arginine (L-NMMA) and its inactive isomer N^G^-monomethyl-d-arginine (d-NMMA) [1] were used. The potency of these compounds in preventing penile erection induced by the above compounds in male rats was correlated with their potency in inhibiting NO synthase, being L-NAME 4–5 times more potent than L-NMMA and D-NMMA ineffective as expected [36,41,77,79,80,81,82,83,84,85]. That the L-NAME prevention of apomorphine-, oxytocin-, NMDA-, 5-HT_1C_ receptor agonists and ACTH-induced penile erection is mediated by the competitive inhibition of NO synthase and not by other effects of this compound was further supported by the ability of i.c.v. l-arginine, the physiological substrate of NO synthase, to prevent the inhibitory effect of L-NAME, in spite of its scarce ability to induce, per se, this behavioral response [86] as discussed below (Section 2.2.1 and Section 2.2.2). Recent studies have shown that L-NAME and S-methyl-thio-citrulline, a selective neuronal NO synthase inhibitor [87] given i.c.v. are also able to prevent penile erection induced not only by apomorphine, oxytocin, and NMDA, but also by other compounds such as hexarelin analogue peptides [88,89,90,91], VGF-derived peptides [92,93] and even when this sexual response was induced by SR 141716A (Rimonabant, [N-(piperidin-1-yl)-5-(4-chlorophenyl)-4-methyl-1H-pyrazole- 3-carboxyamide]), a selective antagonist of the cannabinoid receptor of the CB1 subtype [94], injected into the PVN of male rats [95] (Table 1).

### 2.2. Central NO, PVN and Erectile Function

The major problem with NO synthase inhibitors given systemically is that it is impossible to separate the local (at penile level) and systemic effects (i.e., marked hypotension) from the central effect of these compounds. For this reason, the above studies were soon followed by other studies in which NO synthase inhibitors were injected i.c.v. or into specific brain areas of male rats. These studies revealed that the PVN was one of the brain areas where NO synthase inhibitors act to prevent apomorphine-, oxytocin- and NMDA-induced penile erection and where an increase in the NO content, such as that obtained by injecting the so-called NO donors (drugs that release or produce NO), causes penile erection as will be discussed below [37,86] (see Section 2.2.1 and Section 2.2.2).

#### 2.2.1. NO Synthase Inhibitors Injected into the PVN Abolish/Reduce Drug- and Peptide-Induced Penile Erection in Male Rats

**Figure 2:**
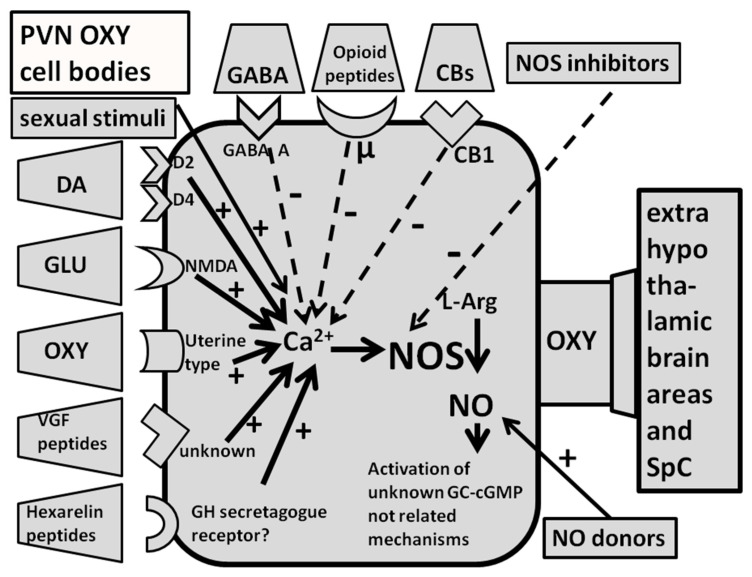
The main mechanism by which dopamine (DA) agonists of the D2 family (D_2_ and D_4_ receptor subtype), oxytocin, NMDA, hexarelin peptides and VGF peptides facilitate penile erection and copulatory activity in male rats is the activation of oxytocinergic neurons originating in the PVN and projecting to the spinal cord and to extrahypothalamic brain areas. Apparently all the above compounds, by acting on their own receptors localized in the cell bodies of these oxytocinergic neurons, increase Ca^2+^ ions influx in the cell bodies of these neurons, in turn causing the activation of NO synthase (NOS), the Ca^2+^ calmodulin-dependent enzyme present in oxytocinergic cell bodies that converts the amino acid l-arginine (l-Arg) to nitric oxide (NO). NO in turn activates oxytocinergic neurons to release oxytocin in the spinal cord and in extrahypothalamic brain areas inducing penile erection and facilitating sexual behavior by a mechanism not involving the guanylate cyclase-cyclic guanosine monophosphate (GC-cGMP) pathway, as discussed below (Section 2.4.2). PVN oxytocinergic neurons facilitate penile erection and sexual activity not only when activated by the above compounds, but also by drugs that increase PVN NO concentration (NO donors), by the blockade of PVN CB1 receptors (which are not located on oxytocinergic cell bodies but increase the activity of the latter ones by increasing glutamatergic neurotransmission in the PVN) (see Section 2.2.4/Figure 3), and by physiological sexual stimuli (e.g., pheromones and others). Conversely, when these oxytocinergic neurons are inhibited, for instance by gamma-amino-butyric acid GABA, opioid peptides/opiate drugs or by drugs that inhibit NOS activity, the spontaneous (i.e., physiologically activated) or drug/neuropeptide-stimulated erectile function and sexual activity is reduced. Details are found in [16,17,40,53,54,55,56,57,73,77,88,89,90,91,92,93,100,101,102,103,104,105,106,107,108,109,110,111,112,113,114,115,116].

As recalled above NO synthase inhibitors were found able to prevent apomorphine-, oxytocin-, and NMDA-, but not 5HT_1C_ or ACTH 1-24-induced penile erection when injected into the PVN of male rats. Indeed L-NAME was found able to prevent this sexual response induced by apomorphine, NMDA and oxytocin not only when injected i.c.v. but also when injected in this hypothalamic nucleus but not in surrounding structures [79,80,81,83,85,86] (Table 1). These findings are in line with the fact that apomorphine, oxytocin and NMDA and other compounds such as hexarelin analogues, VGF related peptides and even SR 141716A, a compound that block cannabinoid receptors of the CB1 subtype, induce penile erection by activating oxytocinergic neurons whose cell bodies are located in the PVN and surrounding area, which project to extra-hypothalamic brain areas and to the spinal cord as discussed below (Figure 2) (Section 2.2.4) [53,54,55,56,57,73]. Conversely, the failure of L-NAME injected into the PVN of male rats to prevent penile erection induced by ACTH 1-24 and 5-HT_1C_ receptor agonists, in spite of its ability to prevent such responses when injected i.c.v., suggest that both these compounds induce penile erection by acting on NO synthase at brain sites different from the PVN with a mechanism that does not involve central oxytocinergic neurotransmission. In line with this possibility, in male rats (i) ACTH 1-24-induced penile erection is not abolished by lesions of the PVN [117], which destroy central oxytocinergic neurons, nor by the blockade of central oxytocinergic receptors with d(CH_2_)_5_Tyr(Me)-Orn^8^-vasotocin, a potent oxytocin receptor antagonist [118], injected into the PVN [119] or by ω-conotoxin, a potent and selective inhibitor of N-type Ca^2+^ channels present mainly in the nervous tissues [120,121] injected in the PVN [102], (ii) ACTH 1-24 induces penile erection when injected in areas surrounding the hypothalamic portion of the third ventricle but not in the PVN [122], and finally (iii) the ACTH symptomatology (which include also stretching and yawning) differs from that induced by dopamine agonists, NMDA and oxytocin, because it begins 25–30 min after the treatment and lasts for several hours [123]. Similarly to that reported above for ACTH 1-24, penile erection induced by 5-HT_1C_ receptor agonists given i.c.v., is not mediated by the activation of central oxytocinergic neurons. Accordingly, the erectile response induced by i.c.v. 5-HT_1C_ receptor agonists is not prevented by the blockade of oxytocinergic receptors, and (ii) 5-HT_1C_ receptor agonists do not induce penile erection when injected in the PVN of male rats [82]. Perhaps more importantly, L-NAME injected into the PVN is also unable to prevent 5-HT_1C_ receptor agonist-induced penile erection [83]. Together the above results suggest that NO synthase inhibitors given i.c.v. prevent penile erection induced by these compounds by acting at sites located downstream to oxytocinergic neurons in a yet undiscovered brain area. In this regard, it is pertinent to recall that a serotoninergic pathway originating in the nucleus paragigantocellularis of the ventral medulla sending projections to the spinal cord in the region of the spinal nucleus of the bulbocavernosus (spinal lumbosacral tract L2-S1) and which inhibits penile erection, has been identified in male rats [124,125,126]. More intriguingly, NO synthase is found localized in neurons of the ventral medulla [127] as well as in the spinal cord [52], raising the possibility that NO acts in these regions to facilitate the pro-erectile effect of these compounds as discussed below (see Section 3).

#### 2.2.2. NO Donors Injected i.c.v. or Directly into the PVN Induce Penile Erection in Male Rats

The studies reviewed above are in line with the hypothesis that NO is involved in the regulation of penile erection induced by dopamine agonists, oxytocin, NMDA, VGF-related peptides and hexarelin analogue peptides at the PVN level, while its involvement in this sexual response induced by ACTH and 5-HT_1C_ agonists seems to occur at sites distinct from the PVN yet to be identified (see above). Further support for the above hypothesis is provided by the results of the studies reviewed in this section, which show that NO donors injected i.c.v. or directly into the PVN of male rats can induce per se spontaneous penile erections indistinguishable from those induced by the drugs and peptides mentioned above. In this regard, it is pertinent to recall that numerous NO donors have been tested and found effective as agents for increasing NO content and causing relaxation of cavernous smooth muscles in in vitro experiments made with isolated strips of cavernous corpora tissue and/or isolated or cultured cavernous corpora cells of several mammals from rat, rabbit, dog and cat to primates and man [128,129,130,131,132,133]. NO donors have also been tested in double-blind crossover trials as a treatment of human erectile dysfunction, but with scarce success [133]. Nonetheless, only a few of the available NO donors have been injected centrally in male rats to test their ability to induce penile erection (Table 2) [103]. These include nitroglycerin, a well-known NO donor clinically used for its potent vasodilating properties and for the treatment of angina pectoris [134], isoamyl nitrite, a NO donor clinically used for the therapy of angina [134] and well known for its aphrodisiac effect [135], sodium nitroprusside, another well-known NO donor clinically used for its potent antihypertensive effect [134], hydroxylamine, which is not used clinically but is converted to NO by various cellular enzymes [24] and S-nitroso-N-acetyl-D-penicillamine (SNAP), which is considered a spontaneous and effective releaser of NO in aqueous solutions [24]. Finally, l-arginine, the physiological substrate of NO synthase and direct precursor of NO, is usually added to the list of NO donors tested for a potential pro-erectile effect. As shown in Table 2, l-arginine, but not d-arginine, injected i.c.v. at doses up to 1000 µg is unable to induce penile erection, but is able to reduce the inhibitory effect of NO synthase inhibitors on apomorphine-, oxytocin- and NMDA-induced penile erection in male rats. Despite its ineffectiveness when given i.c.v., l-arginine, but not d-arginine, is able to induce penile erection when injected into the PVN in more than 70% of the treated rats [86]. These findings confirm that l-arginine-induced penile erection is mediated by its conversion to NO because of the selective substrate stereospecificity of NO synthase (e.g., the l-amino acid only is converted to NO and citrulline). In line with these studies l-arginine (500 nmol/0.5µL) injected into the PVN (i) increases intracavernous blood pressure in male rats kept under pentobarbital anesthesia [43], (ii) increases reflex penile erections in awake rats [42], and (iii) both these responses were abolished by the concomitant administration of L-NAME (500 nmol) [42,43]. At variance from l-arginine, nitroglycerin is able to induce penile erection either when given i.c.v. (33–99 µg) or into the PVN (1.6–6.6 µg) in a dose-dependent manner [37,85]. Like nitroglycerin, isoamyl nitrite is able to induce penile erection immediately after i.c.v. injection at doses between 20 and 100 µg. However, it has not been tested after injection into the PVN because of its incompatibility and/or insolubility with the majority of available solvents [103]. Unlike nitroglycerin and isoamyl nitrite, sodium nitroprusside does not induce penile erection when injected i.c.v. at a dose up to 100 µg, but is able to do so when injected into the PVN at doses higher than 10 µg. The failure of sodium nitroprusside to induce penile erection in male rats when given i.c.v. is probably due to the appearance of collateral behavioral effects, i.e., hyperactivity, hypermotility, sniffing, rearing and convulsions often followed by death within 2–3 h after treatment [103]. These effects mask the expression of penile erection and might be due to the release of highly toxic cyanide ions [136]. Accordingly, despite its ability to induce penile erection when injected into the PVN, sodium nitroprusside has been reported able to reduce sexual competence of male rats during copulatory tests with a receptive female rat when given systemically in a dose-dependent manner, with the highest dose tested (60 µg/kg) found highly toxic and causing high mortality in the treated animals [137]. Like sodium nitroprusside, when injected i.c.v. at doses between 10 and 100 µg hydroxylamine also induces collateral effects such as convulsions that mask the appearance of penile erection in male rats. However, the compound is able to induce penile erection when injected directly into the PVN at doses between 10 and 50 µg, which failed to induce convulsions or other abnormal behaviors [103,113]. Unexpectedly, S-nitroso-D-acetyl-penicillamine is found able to induce dose-dependent gross behavioral changes, e.g., excitation, hypermotility, sniffing and rearing, which mask the expression of penile erection when given i.c.v. at doses up to 100 µg in male rats. The compound is also ineffective when injected into the PVN at the dose of 10 µg. Higher doses have not been tested for the insolubility of the compound in physiological solvents and even in organic solvents (DMSO) [103]. Thus, it is unknown if this NO donor induces penile erection or not in male rats. As recalled above, many other NO donors have been synthesized and a few also tested in in vivo and in in vitro experiments in penile tissues, such as 3-morpholino-sydnonimine hydrochloride (SIN-1) or z-1-[N-(3-ammoniopropyl)-N-(n-propyl)amino]diazen-1-ium-1,2-diolate (PAPA/NONOate), but to our knowledge, not tested at the central level for their effect on penile erection. This is true also for new NO donors under development for the treatment of erectile dysfunction [138,139]. Intriguingly, some of these are also light-controllable, e.g., when administered in the cavernous corpora, they can be activated by light to release NO that becomes immediately available for the activation of guanylate cyclase, thereby increasing cGMP that induces the cavernous corpora relaxation and penile erection [140].

#### 2.2.3. Dopamine Agonists, Oxytocin, NMDA, Hexarelin Analogue Peptides, VGF-Related Peptides Induce Penile Erection by Increasing NO Production in the PVN of Male Rats

The ability of NO synthase inhibitors given into the PVN to prevent apomorphine-, oxytocin-, NMDA-, hexarelin analogue peptide- and VGF derived peptide-induced penile erection in male rats is in line with the hypothesis that the stimulation by the above substances of their own receptors in the PVN is related to a consequent activation of NO synthase in the PVN. Support for this possibility is obtained by measuring the production of NO in the PVN in vivo. This is achieved by measuring the concentration of the reaction products of newly formed NO with O_2_ and H_2_O, NO_2_^−^ and NO_3_^−^ (nitrites and nitrates), which represent an indirect but reliable indicator of NO production in vivo [10,104,142], in the dialysate collected from a vertical microdialysis probe implanted in the PVN of male rats, after the administration of the above substances. As expected, and as summarized in Table 3, apomorphine-, oxytocin-, NMDA-, hexarelin analogue peptide-, VGF derived peptide- and SR 141766A-induced penile erection occur concomitantly to an increase in NO production in the PVN, as demonstrated by the increased concentration of NO_2_^−^ and NO_3_^−^ found in the PVN dialysate [89,90,91,93,96,97,104,143]. The increased NO production induced by the above compounds was reduced/abolished by the prior inhibition of neuronal NO synthase by L-NAME or S-methyl-thio-citrulline given in the PVN of male rats at a dose that also reduces/abolishes the sexual response [89,90,91,93,96,97,104,143]. The prevention/reduction of the increase in NO production and of the concomitant erectile response induced by each one of the above compounds is usually also obtained with the selective blockade of the receptors on which each compound acts in the PVN to increase NO production and penile erection; that is, apomorphine responses are selectively abolished by the blockade of PVN dopaminergic receptors of the D2 family (e.g., receptors of the D_2_ and D_4_ subtype), for instance with haloperidol, sulpiride, cis-flupentixol or L-745,870 (3-[4-(4-chlorophenyl)piperazin-1-ylmethyl]-1H-pyrrolo [2,3-b]pyridine trihydrochloride), a selective D4 receptor antagonist [98,99,104,144,145,146], but not with SCH 23390 [R(+)-7-chloro-8-hydroxy-3-methyl-1-phenyl-2,3,4,5-tetrahydro- 1H-3-benzazepine hydro chloride)], which blocks D1 receptors [97,140], the oxytocin effect by the selective blockade of oxytocinergic receptors with d(CH_2_)_5_Tyr(Me)-Orn^8^-vasotocin, the effect of NMDA by the selective blockade of NMDA receptors with (+)-MK-801 (dizocilpine, [(5R,10S)-(+)-5-methyl-10,11-dihydro-5Hdibenzo-[a,d]cyclohepten-5,10-imine hydrogen maleate]), a potent non-competitive NMDA receptor antagonist [147], but not with CNQX (6-cyano-7-nitro-quinoxaline-2,3-dione), an AMPA receptor antagonist [96,97]. This mechanism cannot be proved for hexarelin analogue peptide- and VGF-related peptide-induced penile erection and the concomitant increase in NO production, as no selective antagonist is available so far of the putative receptors on which these two classes of compounds act, although some evidence for the existence of a receptor specific for hexarelin analogues that mediates penile erection and different from those that mediate growth hormone release induced by these peptides has been obtained in male rats [73,90,91,148]. Conversely, the increase in PVN NO production induced by apomorphine, NMDA, hexarelin analogue peptides and VGF derived peptides is not reduced/abolished by d(CH_2_)_5_Tyr(Me)-Orn^8^-vasotocin given i.c.v. despite its ability to reduce/abolish penile erection induced by these compounds and by oxytocin as well (Figure 2) [88,89,90,91,93,96,97,104,123].

The studies reviewed above show that the activation of NO synthase in the PVN induced by the stimulation of dopamine, oxytocin and NMDA receptors, and possibly other receptors present in the PVN of male rats, leads to penile erection. Perhaps more importantly, the activation of NO synthase takes place in the PVN not only when penile erection is induced by drugs and/or peptides acting in the PVN, but also when this sexual response occurs in physiological contexts, for instance when sexually potent male rats are put in the presence of an inaccessible sexually receptive female rat (e.g., when male rats show non-contact erections) or during copulation, when in copula penile erections occur (Table 4) (see also Section 2.2.3 and Section 3) [54,77,105]. Indeed, an increase in NO production (measured by an increase in the concentration of NO_2_^−^ and NO_3_^−^) is found in the PVN dialysate of sexually potent male rats that show non-contact erections in the presence of an inaccessible receptive female rat and during copulation [77]. That the NO synthase activation and concomitant increase in NO production in the PVN are important for the induction of penile erection in physiological contexts is further supported by the ability of L-NAME, which inhibits NO synthase, injected into the PVN of sexually potent male rats to prevent not only the increase in NO production in the PVN dialysate but also to abolish/reduce non-contact erections and impair copulation with a receptive female rat [77,148].

#### 2.2.4. GABA Agonists, Opioid Peptides/Opiates and Cannabinoids Reduce Spontaneous or Drug- and Peptide-Induced Penile Erection by Inhibiting NO Synthase in the PVN of Male Rats

Changes in the activity of NO synthase in the PVN of male rats also occur when the pro-erectile effect of dopamine agonists, oxytocin, NMDA, hexarelin analogue peptides and VGF-related peptides, on penile erection—or when penile erection occurs in physiological contexts (the presence of an inaccessible receptive female rat and copulation)—is reduced or abolished by drugs that act in the PVN on GABA_A_ receptors, opioid receptors of the µ subtype and cannabinoid receptors of the CB1 subtype. Accordingly, the increase in NO production that occurs during non-contact erections and copulation or when induced by the above drugs and/or peptides given at doses that induce penile erection is reduced/abolished by the injection into the PVN of muscimol, a GABA_A_ receptor agonist, but not by blacofen, a GABA_B_ receptor agonist [105,106,107], by morphine, a µ-opioid receptor agonist, but not by the k-opioid receptor agonist U-69,593 [106,108,109,110,111], given at doses that abolish penile erection without inducing other gross behavioral changes (Figure 2, Table 4 and Table 5). As muscimol effects are abolished by bicuculline, a GABA_A_ receptor antagonist [105,106,107], and morphine effects are abolished by naloxone, a µ-opioid receptor antagonist [77,109,110,111], these results show that muscimol reduces the increase in NO synthase activity that leads to penile erection by stimulating GABA_A_ receptors and morphine by stimulating µ-opioid receptors in the PVN.

**Figure 3:**
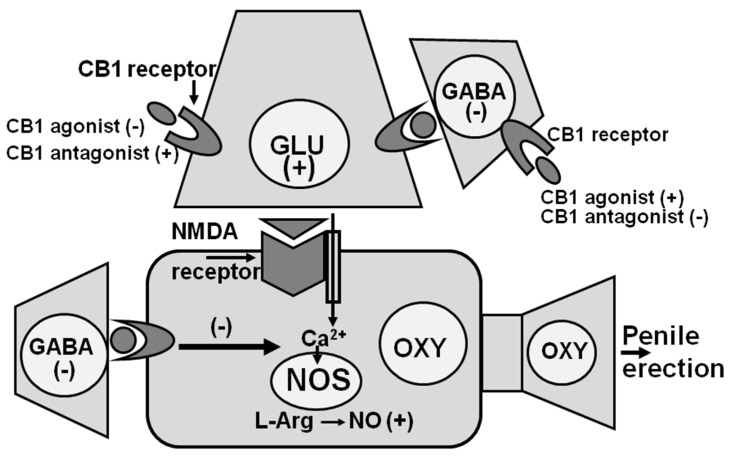
A synthetic representation of two possible mechanisms by means of which endocannabinoids may inhibit penile erection at the PVN level in male rats. Briefly, endocannabinoids inhibit the release of glutamic acid (GLU) from glutamatergic synapses impinging on the cell bodies of oxytocinergic neurons (OXY) by acting mainly on CB1 receptors located on these glutamatergic synapses; alternatively, they can act on CB1 receptors localized on inhibitory GABAergic synapses that impinge on these glutamatergic synapses and whose activation inhibits glutamic acid release. In both cases this causes a reduction in Ca^2+^ influx through Ca^2+^ channels-coupled NMDA receptors in the cell bodies of oxytocinergic neurons mediating penile erection followed by a reduction in NO synthase activity, thereby inhibiting oxytocinergic neurons and penile erection. The above mechanisms may also be not mutually exclusive and operate in concert to inhibit erectile function. Both these mechanisms have been identified in other brain areas rich in CB1 receptors. (+) = activation; (−) = inhibition. Details are found in [95,100,101,112,113].

Changes in NO synthase activity at the level of the PVN are also involved in the inhibitory effect of cannabinoid receptors of the CB1 subtype on penile erection. This became evident when a selective antagonist of the CB1 receptor subtype SR 141716A [94], was found capable of inducing penile erection when injected into the PVN of male rats [95]. Apparently, the blockade of CB1 receptors in the PVN induces penile erection by activating glutamatergic neurotransmission in the PVN. Glutamic acid, in turn, increases Ca^2+^ influx in the cell bodies of PVN oxytocinergic neurons causing the activation of NO synthase and the increase in NO production leading to penile erection (Figure 3). Accordingly, SR 141716A-induced penile erection occurs with an increase in extracellular glutamic acid and NO production in the male rat PVN dialysate [100,101]. Penile erection and the increase in NO production, but not in extracellular glutamic acid induced by SR 141716A, are antagonized by the blockade of NMDA receptors in the PVN with (+)-MK-801, and by the inhibition of NO synthase in the PVN by L-NAME [95,100,101]. In contrast, SR 141716A-induced penile erection, increased NO production and glutamic acid release in the male rat PVN dialysate were all antagonized by morphine [112]. As to the activation of glutamatergic neurotransmission that causes penile erection by stimulating PVN oxytocinergic neurons mediating penile erection secondary to the blockade of CB1 receptors in the PVN, this may be due to CB1 receptors that directly inhibit glutamatergic synapses impinging on oxytocinergic neurons mediating penile erection or to CB1 receptors located in inhibitory GABAergic synapses impinging on glutamatergic synapses and whose blockade decreases the GABA-mediated inhibition of these synapses [100,101,112,113] (Figure 3). Further studies are required to discover if one of the two mechanisms is the most relevant or if the two mechanisms are not mutually exclusive. Irrespective of the exact mechanisms by which cannabinoids inhibit erectile function, this inhibitory effect is mediated at least in part by a reduction in NO activity at PVN level [100,101,112,113].

### 2.3. NO, Erectile Function and Extrahypothalamic Brain Areas

Recent studies have shown that in male rats, NO is involved in the control of erectile function not only at the level of the PVN but also in other brain areas. Among these, the best known are the ventral tegmental area, the posteromedial cortical nucleus of the amygdala, the bed nucleus of the stria terminalis and the spinal cord (Table 6).

In the ventral tegmental area, NO plays a role in the activation of mesolimbic dopaminergic neurons induced by the injection of oxytocin apparently with a mechanism similar only in part to that found in the PVN. Accordingly, the available data suggest that oxytocin injected into the caudal part of the ventral tegmental area of male rats stimulates oxytocinergic receptors located in the cell bodies of mesolimbic dopaminergic neurons. This causes an increased Ca^2+^ influx inside the cell bodies of these dopaminergic neurons, thereby activating neuronal NO synthase. At variance from the PVN, newly formed NO, in turn, activates guanylate cyclase, which is co-localized with NO synthase in the cell bodies of dopaminergic neurons. As discussed below, this increases cGMP levels and activates mesolimbic dopamine neurons to release dopamine in the nucleus accumbens causing penile erection [47,48] (see also Section 2.4.1, Section 2.4.3, Section 3, and Section 3.1.1).

NO is also involved in the induction of penile erection induced by oxytocin injected into the ventral subiculum of the hippocampus, the posteromedial cortical nucleus of the amygdala [49,50,51] and the bed nucleus of the stria terminalis [151,152] of male rats. Accordingly, oxytocin injected into the ventral subiculum and the posteromedial cortical nucleus of the amygdala induces penile erection that occurs with a concomitant activation of NO synthase and an increase in NO production, as measured by the increased NO_2_^−^ and NO_3_^−^ levels in the dialysate obtained from these two brain areas. In the ventral subiculum, newly produced NO acts as an intercellular messenger and activates by a mechanism still unknown excitatory (glutamatergic) efferent, yet to be identified, direct or indirect projections from this area to the ventral tegmental area. Here, the increase in extracellular glutamic acid activates mesolimbic dopaminergic neurons leading to an increased release of dopamine in the nucleus accumbens [49,50,51], as reported above for oxytocin injected into the ventral tegmental area of male rats [47,48]. The activation of NO synthase in the ventral subiculum is necessary for the pro-erectile effect of oxytocin, as this is abolished by NO synthase inhibition. It is likely that a mechanism similar to that described above for the ventral subiculum operates also in the posteromedial cortical nucleus of the amygdala. Oxytocin induces penile erection also when injected in the bed nucleus of the stria terminalis of male rats by increasing extracellular glutamic acid that acts on NMDA receptors located in glutamatergic neurons containing NO synthase, which project from the bed nucleus to the PVN and other extrahypothalamic areas controlling penile erection. As for the ventral subiculum and the posteromedial cortical nucleus, the activation of NO synthase is required for the pro-erectile effect of oxytocin injected in this nucleus, since it is abolished by S-methyl-thio-citrulline injected into the bed nucleus before oxytocin [151,152]. As for the posteromedial cortical nucleus of the amygdala, it is unknown whether NO in this area acts as intercellular or intracellular messenger and if guanylate cyclase is the main target responsible for penile erection induced by the injection of oxytocin.

Finally, NO may also play a role in the control of penile erection at the level of the ventral medulla and the spinal cord. Accordingly, as recalled above (Section 2.1), inhibition by NO synthase by L-NAME given i.c.v. but not in the PVN of male rats prevents penile erection induced by 5-HT_1C_ receptor agonists when injected i.c.v. with a mechanism that does not involve oxytocin [82,83]. This raises the possibility that NO synthase inhibitors prevent penile erection induced by these compounds by acting at sites located downstream to oxytocinergic neurons in a yet undiscovered brain area. In this regard, it is pertinent to recall that NO synthase is found localized in neurons of the ventral medulla [103] as well as in the spinal cord [52] of male rats. Since electrolytic lesions of the nucleus paragigantocellularis of the reticular formation of the ventral medulla facilitate penile reflexes and copulatory behavior in male rats [153] and drugs that enhance 5-HT transmission, especially 5-HT_A_ receptor agonists, impair these sexual responses [154,155,156], it is tempting to speculate that NO synthase inhibitors prevent 5-HT_1C_ receptor agonist-induced penile erection by inhibiting NO synthase in the ventral medulla or in the spinal cord [83].

### 2.4. Facilitation of Erectile Function by Central NO: Mechanism of Action

The studies reviewed above made in male rats show that NO facilitates erectile function at the central level by means of mechanisms which may be different depending on the brain area considered. Among these, the most studied is the activation of central oxytocinergic neurotransmission at the level of the PVN. Here and in other brain areas, numerous experiments aimed at identifying the molecular mechanism activated by NO to facilitate penile erection have been performed, but only in the ventral tegmental area has convincing evidence for a specific mechanism (e.g., activation of guanylate cyclase) been obtained so far. However, some hypotheses on how NO facilitates erectile function when injected into the ventral subiculum have been also proposed, as conducted for the PVN and the ventral tegmental area of male rats where NO acts as an intracellular messenger to facilitate erectile function while it acts as an intercellular messenger in the ventral subiculum.

#### 2.4.1. NO Facilitates Erectile Function in Male Rats by Activating Central Oxytocinergic Neurotransmission

As extensively discussed above, (i) numerous compounds (i.e., dopamine receptor agonists, oxytocin, NMDA, hexarelin analogue peptides, VGF-derived peptides and even the CB1 receptor antagonist SR 141716A) induce penile erection and concomitantly increase NO production in the PVN of male rats; (ii) both these behavioral and biochemical responses are prevented by the inhibition of neuronal NO synthase, which may be direct with non-selective and selective competitive inhibitors, or indirect through the stimulation of GABA_A_, µ-opioid and CB1 cannabinoid receptors in the PVN; (iii) the NO synthase inhibition-induced prevention of drug-and peptide-induced responses is usually reversed by the concomitant administration of l-arginine, the physiological substrate of the enzyme and (iiii) the increase in PVN NO production that occurs during non-contact erection and copulation is antagonized by the inhibition of PVN NO synthase by L-NAME and by the blockade of oxytocin receptors with d(CH_2_)_5_Tyr(Me)-Orn^8^-vasotocin given i.c.v. but not in the PVN (Table 3 and Table 4). These findings provide convincing evidence that in male rats NO plays a key role in the control of erectile function at central level. Such a role is further strengthened by the ability of NO donors injected in the PVN to induce penile erection episodes indistinguishable from those induced by the above substances. More importantly, penile erection induced by oxytocin, dopamine agonists, NMDA, hexarelin analogue peptides and VGF-derived peptides are prevented not only by NO synthase inhibition (see above) but also by the blockade of oxytocinergic receptors with the oxytocin receptor antagonist d(CH_2_)_5_Tyr(Me)-Orn^8^-vasotocin given i.c.v., in line with the hypothesis that these compounds induce penile erection by activating the central oxytocinergic neurotransmission (Figure 2) [53,73]. Since d(CH_2_)_5_Tyr(Me)-Orn^8^-vasotocin given i.c.v. but not in the PVN also antagonizes the pro-erectile effect of NO donors in male rats (Table 7), these findings make it reasonable to assume that the sexual response induced by NO donors injected into the PVN is also mediated by the activation of the same oxytocinergic neurons projecting to extrahypothalamic brain areas, which are activated by dopamine receptor agonists, NMDA, hexarelin analogue peptides, VGF-derived peptides and oxytocin itself. In agreement with this hypothesis, penile erection induced by nitroglycerin, sodium nitroprusside and hydroxylamine injected in the PVN or by isoamyl nitrite given i.c.v. are prevented by d(CH_2_)_5_Tyr(Me)-Orn^8^-vasotocin given i.c.v. but not in the PVN at the dose of 0.1 µg, 10 min before the NO donors [85,103]. Conversely, nitroglycerin-induced penile erections are not prevented by haloperidol (0.5 mg/kg i.p. 30 min before the NO donor), suggesting that the NO donor effect on penile erection is not mediated by dopamine in the PVN or in other brain areas [84]. It is likely that haloperidol does not prevent the pro-erectile response induced by the other NO donors found active in inducing penile erection, but experiments supporting this hypothesis are not available to our knowledge, since no data supporting this hypothesis have been found in the PubMed and Google Scholar medlines made by the authors when writing this review.

#### 2.4.2. Is Guanylate Cyclase the NO Target in the PVN?

**Figure 4:**
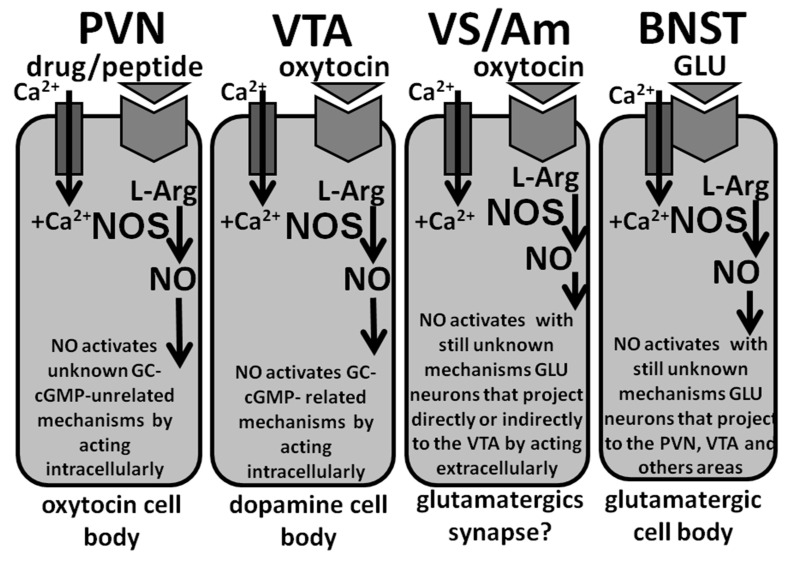
NO influences penile erection by acting in several rat brain areas including the PVN, the ventral tegmental area, the ventral subiculum of the hippocampus, the posteromedial cortical nucleus of the amygdala and the bed nucleus of the stria terminalis. In these areas NO acts by means of different mechanisms. In the PVN, NO activates oxytocinergic neurons projecting to the spinal cord that mediate penile erection and to extrahypothalamic areas including the ventral tegmental area and the posteromedial cortical nucleus of the amygdala. Here, NO acts intracellularly in the neurons in which it is formed by a yet unknown mechanism unrelated to the guanylate cyclase-cGMP pathway. Additionally, in the ventral tegmental area (VTA), NO produced by the activation of NO synthase localized in the cell bodies of mesolimbic-mesocortical dopaminergic neurons acts intracellularly in the dopaminergic neurons in which is produced by the activation of oxytocinergic receptors that induce penile erection. However in these neurons, at variance from the PVN, NO activates guanylate cyclase to increase cGMP levels, which lead to the activation of dopaminergic neurons to release dopamine in the nucleus accumbens and in the medial prefrontal cortex and to induce penile erection. It is likely that a NO-cGMP pathway is also operative in the ventral subiculum (VS) and the posteromedial cortical nucleus of the amygdala (Am) in neurons activated by oxytocin given in these areas at a dose that induces penile erection. At variance from the ventral tegmental area, here NO acts after being released from the neurons in which is formed as an intercellular messenger to activate excitatory neurons projecting directly or indirectly to the ventral tegmental area in order to activate mesolimbic and mesocortical dopaminergic neurons to release dopamine in the nucleus accumbens and in the medial prefrontal cortex and induce penile erection. In the bed nucleus of the stria terminalis (BNST), oxytocin releases glutamic acid that acts on the cell bodies of glutamatergic neurons containing NO synthase and projecting to the PVN, ventral tegmental area, hippocampus and amygdala. How NO formed in the bed nucleus acts to facilitate erectile function is still unknown. Details are found in [47,48,49,50,51,53,54,55,56,57,96,97,98,99,143,144,151,152,160].

The studies reviewed above support the hypothesis that either endogenous NO formed by the stimulation of dopamine, NMDA, oxytocin, hexarelin analogue peptide and VGF-derived peptide receptors in the PVN, or by NO donors injected in the PVN, activates oxytocinergic transmission to induce penile erection in male rats. Unfortunately, these studies do not provide any evidence about the mechanism by means of which NO activates oxytocinergic neurons. However, these studies suggest that guanylate cyclase, one of the best-known targets of NO in peripheral tissues and in several brain areas as well [5,11,12,25,157] is not involved in the facilitatory effect of NO on erectile function in the PVN. Accordingly, the microinjection into the PVN of methylene blue and LY 83583 [6-(phenylamino)-5,6-quinoline-dione], two putative inhibitors of this enzyme [158,159] at doses between 5–20 µg are unable to prevent penile erection induced by apomorphine, oxytocin, NMDA or NO donors in male rats (Table 8) [79,80,85,103]. This suggests that NO acts in the PVN on other targets in order to activate oxytocinergic neurons projecting to extra-hypothalamic brain areas and the spinal cord mediating penile erection. In this regard it is pertinent to recall that NO might interact with numerous other enzymes that, like guanylate cyclase, bind metal ions such iron, and that other targets of NO, such as cellular ADP-ribosyl-transferases, have been identified (reviews on other targets of NO are found in [4,12,26]). However, this interpretation is complicated in part by the ability of both methylene blue (100–400 µg) and LY 83583 (100–200 µg) to prevent penile erection induced by apomorphine, oxytocin, NMDA, NO donors and 5HT_1C_ receptor agonists when injected i.c.v. in male rats (Table 8) [79,80,85,103]. One possible explanation for this discrepancy is that cGMP is involved in the expression of penile erection induced by the above substances in some yet undiscovered brain area distant from the PVN. In agreement with this possibility, methylene blue given i.c.v. is unable to prevent the increase in NO production in the PVN induced by apomorphine, oxytocin and NMDA despite its ability to prevent penile erection (Table 8). That guanylate cyclase might not be the target of NO in the PVN for the induction of penile erection is also suggested by experiments showing that (i) 8-Bromo-cGMP, an active and stable (phosphodiesterase-resistant) cGMP analog that would be expected to mimic the effect of endogenous cGMP, is unable to induce penile erection when injected into the PVN at doses between 0.1 and 50 µg in male rats [96,103,104,143,160], and (ii) methylene blue injected into the PVN is unable to reduce/abolish both non-contact erections in sexually potent male rats put into the presence of an inaccessible sexually receptive female rats and copulation when in copula erections take place and the concomitant increase in NO production that occurs in the PVN in these physiological contexts (Table 4) [76] (see also Section 2.2.3 and Section 3). In this regard, it must be noted that a NO-cGMP signaling pathway might not exist or be active in the PVN of male rats, although such pathway has been well characterized not only by biochemical but also by immunocytochemical studies in other brain areas, such as the hippocampus and the cerebellum [25]. In agreement with this hypothesis, the PVN of male rats contains only very low amounts of guanylate cyclase immunoreactivity [25] and PVN oxytocinergic neurons, labeled by NO synthase-directed antibodies, are not labeled by guanylate cyclase-directed antibodies [29]. Irrespective of the mechanism by which NO acts in the PVN to activate central oxytocinergic neurotransmission, as will be discussed below, together with other findings these results also support the hypothesis that NO facilitates penile erection by acting as an intracellular messenger in the male rat PVN (Figure 4) (see Section 2.4.4).

#### 2.4.3. Guanylate Cyclase Is the NO Target in the Ventral Tegmental Area of Male Rats

At variance from the PVN, the available data suggest that NO, produced by the activation of NO synthase located in the cell bodies of mesolimbic dopaminergic neurons by the increased Ca^2+^ influx that takes place after activation of oxytocinergic receptors expressed in these cell bodies by oxytocin, activates guanylate cyclase in the ventral tegmental area of male rats in order to facilitate penile erection induced by oxytocin injected into this mesencephalic area [47,48] (Table 9). In agreement with this hypothesis, (i) the erectile response induced by oxytocin injected into the male rat caudal ventral tegmental area occurs concomitantly to an increased NO production and both behavioral and neurochemical response are antagonized not only by the blockade of oxytocinergic receptors by d(CH_2_)_5_Tyr(Me)-Orn^8^-vasotocin but also by the neuronal NO synthase inhibitor S-methyl-thio-citrulline injected into the caudal ventral tegmental area before oxytocin [47,160]; (ii) the erectile response but not the increase in NO production is also antagonized by ODQ (1H-[1,2,4]oxadiazole[4,3-a]quinoxalin-1-one), a potent inhibitor of guanylate cyclase [25], injected into the caudal ventral tegmental area before oxytocin [47,160]; (iii) 8-bromo-cGMP induces penile erection episodes (indistinguishable from those induced by oxytocin) when injected into the caudal ventral tegmental area of male rats with a U-inverted dose response curve, being the maximum response found at the dose of 3 µg [47,48]. Further support for the involvement of the NO-cGMP signaling pathway in the induction of penile erection by oxytocin in the ventral tegmental area of male rats comes from immunohistochemistry experiments showing that the ventral tegmental area contains mesolimbic dopaminergic cell bodies that are labeled for both NO synthase and guanylate cyclase (the two enzymes are located in the same cell body), and which run close to oxytocinergic fibers originating from the PVN [47]. These results support the hypothesis that NO synthase and guanylate cyclase co-exist in the cell bodies of mesolimbic dopaminergic neurons whose activation facilitates penile erection. In agreement with this hypothesis, experimental evidence showing that NO synthase and guanylate cyclase may also coexist in specific neuronal populations is also available, especially in mesencephalic structures, favoring an intracellular autocrine role of a NO-cGMP pathway in these neurons [161] (Figure 4). This is in spite of the fact that NO synthase and guanylate cyclase are often found in neurons juxtaposed with NO synthase in the cell bodies of and guanylate cyclase in nerve endings of excitatory (often glutamatergic) neurons that impinge on the NO synthase containing cell bodies in many parts of the CNS [6,12,25]. In these areas NO is commonly accepted to act as a retrograde messenger molecule in many neural processes mainly related to long-term potentiation and/or depression [162]. Together with the inability of the NO scavenger hemoglobin to inhibit penile erection despite its ability to reduce NO_2_ concentration in the dialysate obtained from the caudal ventral tegmental area of male rats, these findings confirm that NO acts mainly as an intracellular messenger in the ventral tegmental area, as it will be discussed below (see Section 2.4.4). That cGMP may have a role in facilitating erectile function at level of the ventral tegmental area is also supported by the ability of sildenafil and vardenafil, two orally active phosphodiesterase type 5 inhibitors clinically used for the therapy of erectile dysfunction, to increase the number of non-contact erections in rats screened for showing or not this sexual response compared to control rats when injected directly into the ventral tegmental area [163]. Accordingly, the two drugs are expected to increase cGMP levels in the ventral tegmental area (due to the inhibition of cGMP degradation by phosphodiesterase type V). Interestingly, in this physiological context, the increase in the number of non-contact erections takes place concomitantly to an increase in extracellular dopamine in the dialysate obtained from the nucleus accumbens of these rats [163], as found with oxytocin injected into the VTA [47,48].

A NO-cGMP signaling pathway might play a role in the induction of penile erection by oxytocin injected into the ventral subiculum or into the posteromedial cortical nucleus of the amygdala of male rats. In fact, oxytocin injected in these two areas induces penile erection [49] and increases NO production [50] as found in the ventral tegmental area. Both these responses are antagonized by the oxytocin receptor antagonist d(CH_2_)_5_Tyr(Me)-Orn^8^-vasotocin and by the NO synthase inhibitor S-methyl-thio-citrulline [50]. Apparently, oxytocin injected into the ventral subiculum induces penile erection by acting on its own receptors localized in yet unidentified neurons whose activity when changed by the stimulation of oxytocin receptors causes an increase in glutamic acid neurotransmission (Figure 4). Accordingly, an increase in extracellular glutamic acid is found in the dialysate from the ventral subiculum of male rats 15 min after the oxytocin injection and usually 15 min before the appearance of penile erection, which instead occurs 25–30 min after oxytocin injection. Glutamic acid in turn causes penile erection, possibly activating excitatory (possibly glutamatergic) efferents projecting from the ventral subiculum to extra-hippocampal areas such as the medial prefrontal cortex. This leads to the activation of glutamatergic neurons projecting from the prefrontal cortex to the ventral tegmental area, in turn activating mesolimbic mesocortical dopaminergic neurons and penile erection ([50,51] and references therein). Alternatively, glutamic acid in the ventral subiculum might activate glutamatergic neurons projecting directly to the ventral tegmental area, since in male rats a direct glutamatergic pathway from the ventral subiculum to the ventral tegmental area, which activates mesolimbic dopaminergic neurons, cannot be ruled out ([50,51] and references therein). It is likely that the neurons expressing oxytocin receptors also contain NO synthase whose activation leads to an increase in NO that, when produced, activates glutamatergic neurons projecting to the medial prefrontal cortex or the ventral tegmental area with a still unknown mechanism. Accordingly, since hemoglobin, a NO scavenger, injected into the ventral subiculum before oxytocin prevents both the increase in NO production and penile erection in male rats, it is also likely that newly formed NO travels intercellularly to reach glutamatergic neurons that, once activated, lead to penile erection mediated by the increased glutamatergic activity in the ventral tegmental area followed by that of mesolimbic dopaminergic neurons [50,51] (see Section 2.4.4). Further experiments are required to verify if guanylate cyclase is involved in the facilitatory effect of oxytocin injected in the ventral subiculum and to ascertain if mechanisms similar to those summarized above are also operative in the posteromedial cortical nucleus of the amygdala and in the bed nucleus of the stria terminalis of male rats after the injection of oxytocin at doses that increase NO production and induce penile erection [49,151,152].

#### 2.4.4. NO Facilitates Erectile Function in Male Rats by Acting Intracellularly in the PVN and in the Ventral Tegmental Area and Intercellularly in the Ventral Subiculum

The studies reviewed above performed on male rats also show that NO may act as an intracellular or intercellular messenger depending on the brain area where it acts to control erectile function. As to the PVN and the ventral tegmental area, the available data support a role for NO as an intracellular messenger (Figure 4). Accordingly, hemoglobin, a potent NO scavenger [164] injected either i.c.v. (100–400 µg) or in the PVN (10–20 µg), is unable to prevent penile erection induced by oxytocin, apomorphine, NMDA, NO donors or 5 HT_1C_ agonists injected into the PVN (Table 8) [79,80,81,103] or by oxytocin injected into the caudal ventral tegmental area [47]. This occurs in spite of the ability of hemoglobin-injected i.c.v. or in the PVN to abolish/reduce the increase in NO metabolites in the male rat PVN extracellular dialysate induced by the above substances [79,80,81,96,104] or when this increase occurs in physiological contexts, [e.g., in the presence of a sexually receptive female rat (non-contact erections) or during copulation] [77] (Table 4), or when induced by oxytocin injected into the caudal ventral tegmental area as well [47] (Table 9). Although these findings may be considered as evidence against the role of guanylate cyclase in the control of erectile function in the PVN at variance from the ventral tegmental area, they are far from being decisive since hemoglobin, due to its high molecular weight, would bind NO exclusively in the extracellular space, being unable to cross cellular membranes. Hence, the inability of hemoglobin to prevent penile erection induced by the above substances including oxytocin when injected into the PVN, or when this sexual response occurs in physiological contexts, which all increase NO production in the PVN of male rats, may be explained by assuming that NO is acting intracellularly in those neurons in which it is formed to induce penile erection rather than after having been released in the extracellular space to reach other neuronal targets in these two brain areas (Table 4, Table 8 and Table 9). However, this assumption does not rule out that NO might act also as an intercellular messenger in the PVN and in the caudal ventral tegmental area and perhaps also in the other brain areas considered in this review, where the injection of oxytocin induces penile erection by activating NO synthase and increasing NO production. Indeed, irrespective of the brain area considered, NO released out from the neurons in which it is formed and escaped to the scavenging effect of hemoglobin, might well act on and mediate other effects by acting on neurons close to those from which it has been released.

At variance from the PVN and the ventral tegmental area, in the ventral subiculum of the hippocampus NO acts as an intercellular messenger to facilitate penile erection induced by oxytocin injected in this area (Figure 4). Accordingly, in male rats hemoglobin injected into the ventral subiculum before oxytocin prevents both the increase in NO production and penile erection (Table 9). This suggests that once produced by the stimulation of oxytocinergic receptors, NO travels intercellularly to reach glutamatergic neurons that, once activated, lead to penile erection mediated by the increased glutamatergic activity in the ventral tegmental area. Unfortunately, in the experiments summarized above [50,51], the effect of a guanylate cyclase inhibitor injected into the ventral subiculum or of 8-Bromo-cGMP, which would mimic the effect of endogenous cGMP, was not tested in male rats to ascertain if NO was acting on guanylate cyclase to activate the glutamatergic neurons, which are proposed to activate glutamic acid neurotransmission in the ventral tegmental area for inducing penile erection as discussed above. Further experiments are required to verify if NO acts as an intracellular or intercellular messenger in the posteromedial cortical nucleus of the amygdala and in the bed nucleus of the stria terminalis after the injection of oxytocin at doses that induce penile erection and increase NO production [49,151,152].

## 3. Central NO and Male Sexual Behavior

It is well known that sexual behavior plays a key role in the reproduction of all living animals, from insects to mammals, including humans. As already recalled in Section 2, in male mammals sexual behavior is testosterone dependent and is organized in two main phases, anticipatory and consummatory, and several well-defined and quantifiable parameters have been identified in each phase and in both males and females. The studies on male sexual behavior have usually been and are still performed mainly in rats because of their availability and the well-characterized sequence of copulatory behavior and its parameters [59,60,165,166]. However, data on male sexual behavior in other animal species (mice, rabbits, hamsters, voles, primates, and even birds, as the Japanese quail and the zebra finch) are also available [61,167,168,169,170,171,172]. Penile erection, seminal emission and ejaculation are the main components of the consummatory phase of the male sexual response and are preceded by an anticipatory phase, which includes motivation towards and searching for an adequate partner for copulation [59,60,173]. Briefly, when sexual (visual, auditory, olfactory, tactile and even imaginative in humans) stimuli reach the central nervous system, neural pathways are activated which convey sexual information from the higher brain centers through the spinal cord and the autonomous nervous system to the genital apparatus to induce penile erection, thus allowing copulation that will culminate with ejaculation with the female [16,18,59,60,61,75,173]. It is well known that numerous neurotransmitters and neuropeptides are involved at the central and peripheral level in the control of both phases of male sexual behavior. Among neurotransmitters, the best known are dopamine, serotonin, glutamic acid and gamma-amino-butyric acid (GABA) [40,53,56,63,70,73,114,115]. They influence sexual behavior by acting in different brain areas, from the hypothalamus and its nuclei (e.g., PVN and lateral hypothalamus), to the medial preoptic area and other brain areas, which include the ventral tegmental area, the nucleus accumbens, the prefrontal cortex, the hippocampus, the amygdala, the bed nucleus of the stria terminalis, the medulla oblongata and the spinal cord. In these brain areas neurotransmitters often interact in a concerted manner with each other and/or with neuropeptides, such as oxytocin, adrenocorticotropin (ACTH), α-melanocyte stimulating hormone (α-MSH) and opioid peptides in the control of several aspects of sexual behavior, from sexual motivation and arousal to sexual performance or both [53,55,56,57,66,73,102,116,149,150,174]. As recalled above, NO was added to the list of neurotransmitters/neuromodulators that play a role in sexual behavior in 1990s when it was discovered that this molecule formed from l-arginine by NO synthase is the physiological neurotransmitter of penile erection at local level (see Section 1). This is due to the key role of NO in the activation of guanylate cyclase and the consequent increase in cGMP levels that are responsible for the relaxation of the cavernous corpora smooth muscles allowing penile erection to occur (see Section 1). This discovery was soon followed by studies showing that NO plays a role in erectile function (discussed above) and sexual behavior also at the central level [37,38,41]. The medial preoptic area and the PVN have been identified as the two main brain sites where NO acts to facilitate male sexual behavior, although some experimental evidence for the involvement of the ventral tegmental area is also available.

### 3.1. Medial Preoptic Area

As found with erectile function, the first evidence for a facilitatory role of NO in sexual behavior was obtained with the NO synthase inhibitor L-NAME given intraperitoneally, which was found able to impair copulatory behavior in a dose dependent manner in male rats, while l-arginine induced only modest effects [38,41,175]. The inhibitory effect of L-NAME was found much more marked after i.c.v. injection, being the drug found able to completely prevent ejaculation in sexually naive rats, but not in sexually experienced rats [41]. The latter study also reported that in the PVN, but not in the amygdala or the bed nucleus of the stria terminalis, of sexually potent male rats, NO synthase messenger RNA content was twice that found in sexually impotent rats (e.g., rats that do not copulate when put together with a sexually receptive female rat). These findings led us to suggest that NO may facilitate copulatory behavior in male rats by acting not only at penile level but also at the central level and in particular at the level of the PVN. The medial preoptic area was soon identified as one of the brain areas where central NO may facilitate copulatory behavior (Table 7). In this regard it is pertinent to recall that the medial preoptic area plays a key role in sexual behavior and that several neurotransmitters interact to control penile reflexes and copulation at this level [59,60]. Among these, the most studied are dopamine, serotonin and glutamic acid [176,177]. In particular, it is well known that dopamine is released in this area during copulation and that dopamine receptor agonists infused into the medial preoptic area facilitate, and antagonists impair, copulatory behavior in male rats [178,179]. More important for this review, (i) the administration of l-arginine (e.g., the NO precursor) also increases dopamine release in the medial preoptic area, (ii) the infusion of L-NAME at the concentration of 400 mM in the dialysis buffer via the microdialysis probe, but not its inactive isomer D-NAME into the medial preoptic area for 3 h prior to the introduction of a sexually receptive female rat in the mating arena, antagonized the extracellular dopamine increase that occurs in the medial preoptic area during copulation and impairs copulation [180]. This led to suggest that NO in the medial preoptic area facilitates copulation by increasing dopamine release in male rats (Table 10).

A facilitatory role of NO in sexual behavior has been also reported to occur in the medial preoptic area of male mice [186], although some controversy exists on this point [187], in line with the existence of differences in sexual behavior and its neural control between male mice and rats (see Section 1).

#### 3.1.1. NO Facilitates Erectile Function and Sexual Behavior by Acting Intercellularly as a Retrograde Messenger in the Medial Preoptic Area of Male Rats

**Figure 5:**
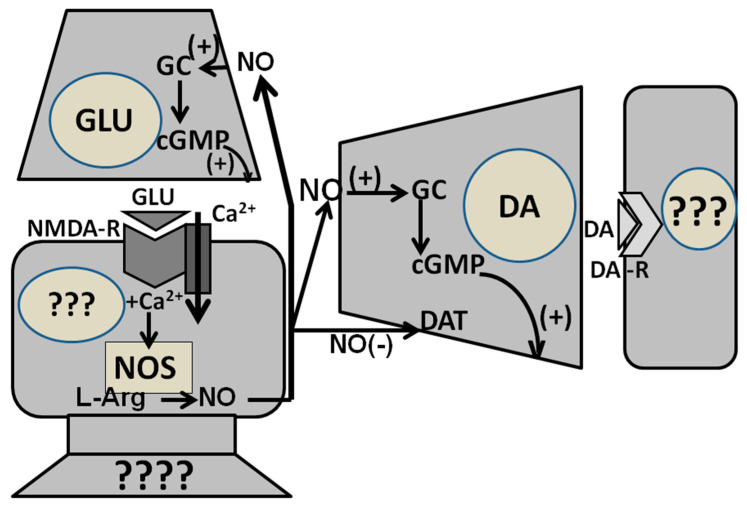
A hypothetical mechanism to explain how NO facilitates penile reflexes and copulation in male rats by increasing dopamine release in the medial preoptic area induced by glutamic acid released from glutamatergic synapses of neurons originating in the amygdala and in the bed nucleus of the stria terminalis activated by sexual stimuli (e.g., those produced by a sexually receptive female rat) and controlling dopamine release from incertohypothalamic dopaminergic neurons. Briefly, NO—once formed by yet unidentified neurons/cells in the medial preoptic area—is released in the extracellular space and acts as a retrograde intercellular messenger on (i) dopaminergic synapses to potentiate dopamine release and facilitate copulation, and (ii) glutamatergic synapses to sustain glutamic acid release facilitating dopamine release, by activating a NO-cGMP signaling pathway. NO may also facilitate dopamine release from dopaminergic synapses by inhibiting the dopamine transporter (DAT), thus allowing dopamine to persist in the extracellular space and act for longer times on its receptors. (+) = activation; (-) = inhibition; (???) = unknown neurotransmitters. Details are found in [38,39,44,45,175,176,177,178,179,180,181,182,183,184,185].

The discovery that NO facilitates copulation by increasing dopamine release in the medial preoptic area was soon followed by numerous studies aimed at identifying the mechanism by which NO induces this effect. These studies show that NO facilitates penile reflexes (e.g., penile erections induced by simulation of the genitalia) and dopamine release in the medial preoptic area of male rats by acting as a retrograde messenger to increase glutamic acid neurotransmission, which is responsible for the increase in dopamine release induced by copulation [178,181,182]. In fact, it is generally accepted that the increase in extracellular dopamine that occurs in the medial preoptic area during sexual activity is mediated by glutamic acid released from neuronal efferents originating in the medial amygdala and the bed nucleus of the stria terminalis, which are activated by sexual mainly olfactory (pheromones and others) stimuli [177]. However, how glutamic acid released in the medial preoptic area stimulates dopamine release from the dopaminergic nerve endings of incertohypothalamic neurons is not completely understood. Experimental evidence shows that glutamic acid acts on NMDA receptors coupled to voltage-dependent Ca^2+^ channels in medial preoptic yet to be identified cells containing NO synthase. The NMDA receptor-coupled voltage-dependent Ca^2+^ channels are linked through their carboxy-terminal tail via a PSD-95 protein–protein interaction domain to NO synthase [188], thereby coupling NO synthase with the NMDA receptor (a review on this coupling in the medial preoptic area is found in [179]). The mechanisms by which NO facilitates dopamine release from incertohypothalamic dopaminergic nerve endings is unknown. One possibility is that NO synthase, once activated by the increased Ca^2+^ influx, increases the production of NO, which is released in the extracellular space and travels as a retrograde messenger to dopaminergic nerve endings. Here the available data suggest the involvement of a NO-cGMP pathway, e.g., that NO activates guanylate cyclase, which in turn increases cGMP levels to sustain dopamine release and copulation. Accordingly, the infusion by reverse dialysis of 8-Bromo-cGMP, which mimics cGMP, increases, and of ODQ, which inhibits guanylate cyclase, decreases extracellular dopamine in the medial preoptic area, respectively. ODQ also blocked the increase in extracellular dopamine levels induced by the NO donor sodium nitroprusside, while the competitive NO synthase inhibitor L-NMMA was ineffective in blocking the 8-Bromo-cGMP-induced increase in dopamine, in line with a site of action for cGMP located downstream to NO. Finally, 8-Bromo-cGMP facilitated, while ODQ inhibited copulation of male rats with a sexually receptive female rat (Table 10) (Figure 5) [182]. However, other actions of NO released in the extracellular space in addition to those recalled above cannot be ruled out. For instance, NO might also facilitate dopamine release by inhibiting the dopamine transporter (DAT) on dopaminergic nerve endings, contributing to a further increase in dopamine content in the extracellular space and to prolong its action on dopaminergic receptors [178]. Moreover, NO might also travel back to glutamatergic synapses and act there to sustain glutamic acid release, possibly by activating a NO-cGMP signaling pathway similar to that described above in the dopaminergic nerve endings. The two mechanisms might also be not mutually exclusive and co-operate both to maintain dopamine release and copulation. Further studies are necessary to verify this possibility.

### 3.2. PVN

The medial preoptic area is not the only brain area in which increased NO activity has been demonstrated during sexual behavior in male rats. Experimental evidence shows that this also takes place in the PVN. In fact, as recalled above, (i) in the PVN, but not in the amygdala and in the bed nucleus of the stria terminalis (two areas involved in sexual behavior) sexually potent male rats have NO synthase messenger RNA levels twice higher than those found in sexually impotent rats (e.g., rats that do not copulate when put together with a sexually receptive female rat) [41]; (ii) neuronal NOS protein expression measured by Western blot analysis is reduced by about 40% in the PVN of streptozocin-treated rats (a rat model of diabetes) that show a blunted erectile response to NMDA injected into the PVN compared control rats, which is restored by restoring neuronal NOS within the PVN by gene transfer using adenoviral transfection [189] and (iii) increased NO production also occurs in the PVN of male rats when penile erection takes place in physiological contexts, e.g., when male rats are put in the presence of an inaccessible receptive female rat and during copulation [74]. The latter behavioral and biochemical effects are prevented by the inhibition of NO synthase in the PVN (Table 4) [47,48]. However, at variance from the medial preoptic area, in the PVN NO acts intracellularly to facilitate non-contact erections and copulation and with a mechanism yet to be identified that does not involve a NO-cGMP pathway. Accordingly, 8-Bromo-cGMP injected into the PVN does not induce penile erection and inhibition of guanylate cyclase by methylene blue or NO scavenging from the extracellular space in the PVN with hemoglobin does not impair non-contact erections and copulation (Table 4, Figure 2) [47,48]. Further studies are necessary to identify the mechanism by which NO activates oxytocinergic neurotransmission in the PVN to facilitate non-contact erections and copulation in male rats.

### 3.3. Ventral Tegmental Area, Ventral Subiculum, Amygdala and Bed Nucleus of the Stria Terminalis

To our knowledge, no data are available showing that increased NO production also occurs in the ventral tegmental area, ventral subiculum, amygdala and bed nucleus of the stria terminalis during sexual behavior despite the increase in NO production that occurs in these areas after the injection of oxytocin at a dose that induces penile erection; an increase that is prevented by the inhibition of NO synthase in these areas. However, a little experimental evidence supporting that this may occur in the ventral tegmental area is available. Accordingly, sildenafil and tadalafil, two phosphodiesterase type V inhibitors (clinically used for the therapy of erectile dysfunction) injected into the ventral tegmental area increase the number of non-contact erections in both groups of male rats that have been previously screened for their ability to show or not, respectively, this sexual response [163]. Interestingly, the facilitatory effect on non-contact erection by these drugs takes place with a concomitant increase in extracellular dopamine in the nucleus accumbens dialysate, the dopamine increase being higher in male rats screened for not showing non-contact erections than in those showing the sexual responses [163]. This finding is in line with the fact that these compounds injected in the ventral tegmental area are expected to increase the endogenous cGMP levels due to their ability to markedly and selectively inhibit phosphodiesterase activity that inactivates cGMP. It is tempting to speculate that an increase in NO production similar to that found after oxytocin injection in the ventral tegmental area at a dose that induces penile erection in male rats [47,48] also occurs in this area when penile erection occurs in physiological contexts (i.e., presence of an inaccessible receptive female rat and during copulation). Further experiments are necessary to verify this hypothesis.

## 4. Central NO and Female Sexual Behavior

As already described for male sexual behavior in Section 3, female sexual behavior is also organized in an anticipatory phase, which includes motivation towards and searching for an adequate partner for copulation [59,60,166,173], and a consummatory phase, whose main components include the manifestation of lordosis (see below), vaginal lubrication, clitoris erection and possibly orgasm in the majority of female mammals [190], except that human primates, including women, do not show lordosis during sexual intercourse. Female sexual behavior also has been extensively studied in female rats (although studies on other rodents and animal species are available). Briefly, in female rats, the anticipatory phase is characterized by proceptive behaviors such as hops/darts and solicitation episodes, while the consummatory phase is characterized mainly by lordosis, i.e., the assumption of a posture characterized by arching of the back and lateral moving of the tail, which occurs when the male touches the flanks and/or perineal region of the female and which is aimed to facilitate intromission of the male penis into the vagina for ejaculation [166,173]. Most important, lordosis occurs only when female rats are sexually receptive, that is when they are in the estrous phase of the estrous cycle, and is strictly dependent from the marked increase in circulating estradiol that occurs in this phase of the cycle. In fact, at variance from male rats, female rat sexual behavior is under the control of the cyclic variations of ovarian hormones, estradiol and progesterone, which allow female rats to become sexually receptive and able to engage in copulation with a male only after the increase in estradiol levels ([191] and references therein). In general, female sexual responses are activated and mediated by the same neural circuitry shown schematically in Figure 1 [192], although it has to be integrated with the neural pathways that lead to the lordotic posture only when the male touches the flanks and/or perineal region of a receptive female. Briefly, the lordosis reflex is activated by the sensory tactile stimuli secondary to the touch of female perineal regions, which send sensory inputs that travel in spinal nerves to the lumbosacral L1-S1 spinal tract, and from here to the mesencephalic periaqueductal gray matter. This brain region coordinates striated back muscles activity together with other brain regions and in particular with the sexually dimorphic ventromedial nucleus of the hypothalamus, which usually inhibits the lordosis reflex, except when circulating ovarian estradiol levels are increased, as it occurs in sexually receptive females in the estrous phase of the cycle [191,193,194,195] and Section 6).

As already reported for male sexual behavior, numerous neurotransmitters and neuropeptides are also involved in female sexual behavior and NO was only added to this list in 1995. However, at variance from male sexual behavior, the role of central NO in female sexual behavior has received much less attention, as revealed by the much lower number of studies on NO and female sexual behavior compared to those of NO and male sexual behavior that appear in Pub Med and Google Scholar medlines. These earlier studies show that neurons containing NO synthase in the rat hypothalamus stimulate the pulsatile release of LH-RH in vivo and in vitro, which also stimulate female sexual behavior as measured by the lordotic posture assumed by the female when the male touches the female rat’s flanks, [196,197]. In this study L-NAME, but not D-NAME, injected into the third cerebral ventricle was found able to reduce and sodium nitroprusside to increase the number of lordosis episodes induced by progesterone when administered to estrogen-primed ovariectomized rats. Since sodium nitroprusside also facilitates lordosis in the absence of progesterone and this response is blocked by a LH-RH antiserum, this leads to suggest that progesterone causes the release of NO, which in turn stimulates LH-RH release that facilitates lordosis and sexual behavior in female rats [196,197,198]. The facilitatory effect of NO on lordosis behavior is apparently mediated by a NO-cGMP pathway, since inhibition of guanylate cyclase by ODQ given into the third ventricle significantly decreased lordosis quotient in ovariectomized rats primed with estradiol and progesterone [199]. One of the hypothalamic areas in which NO may act to facilitate lordosis is the ventromedial nucleus, as suggested by the increase in neuronal NO synthase mRNA that occurs in this but not in the arcuate or supraoptic nucleus following short-term estrogen treatment [200]. Recent studies in female mice suggest that NO synthase containing neurons in the ventrolateral part of the ventromedial nucleus modulating lordosis, but not mating preference, may be under the control of kisspeptin neurons originating in the hypothalamic anteroventral periventricular area [201,202]. NO seems also play a role in the higher expression of NO synthase that occur in the medial preoptic area of sexually experienced female rats in a paced mating test with a male rat when compared to naive female rats [203]. Whether mechanisms similar to those identified in female rats and mice are operative also in the central nervous system in women is unknown, although it is tempting to speculate that this preclinical evidence may prove useful for the therapy of female sexual dysfunctions [141].

## 5. Can Central NO Have a Role in Strategies Aimed to Improve Erectile Function and Sexual Behavior in Humans?

The studies reviewed so far show that central NO is involved in the control of erectile function and sexual behavior at several levels, from the PVN and the medial preoptic area to the limbic system and the spinal cord. However, it is difficult sustaining that the facilitatory role of this neurotransmitter/neuromodulator may be selectively activated at the central level to improve penile erection in men with erectile dysfunction of central origin (psychogenic erectile dysfunction) either physiologically or pharmacologically. This is due to the main role of NO at the penile level in the relaxation of cavernous corpora smooth muscles. In fact, such selectivity is difficult to obtain, as this requires the use of an administration route yet to be discovered that allows a drug (such as a NO synthase inhibitor or a NO donor) to cross the blood–brain barrier and reach the central nervous system without reaching the systemic blood circulation, or the synthesis of drugs that differentially release NO in the brain, cavernous corpora and vascular tissues. This is a very important point because NO is one of the main vasorelaxing modulators present in all vascular tissues, and this may cause severe complications (e.g., marked hypotension). The achievement of a selective action at central level is also very important because penile neuronal NO synthase, its variants and its regulatory protein inhibitor are present and co-localized not only at the local level in penile nerves and pelvic ganglia, but also in the hypothalamic (PVN and medial preoptic area) and spinal cord regions involved in the control of penile erection [204]. Irrespective of the above criticism, several studies investigated the use of compounds that are supposed to increase NO levels or activate NO synthase to improve erectile function in men. These compounds include EGb 761 (a Ginkgo biloba extract), ginsenoside Rg1 (isolated from Panax ginseng) and Lycium Barbarum Polysaccharide, obtained by the Lycium Barbarum berries extensively used in Chinese medicine, and are used as food supplements. In rats these compounds have been found able to improve non-contact erections and sexual behavior, sometimes accompanied by increases in NO synthase expression in the hypothalamus, the medial preoptic area and the spinal cord [205,206,207]. However, the most widely used food supplement by men seeking natural treatment and/or self-medication for erectile dysfunction is l-arginine, the physiological NO synthase substrate. Several studies show an improving effect of l-arginine on erectile function in men. In a recent study, l-arginine, given daily at the dose 2.500 mg for 12 weeks, was found able to improve erectile function in men with low to moderate, but not a severe grade of erectile dysfunction, as determined by the International Index of Erectile Function-Erectile Function (IIEF-EF) questionnaire and in particular from the change in IIEF-EF score and in per-patient percentage of “yes” responses to the Sexual Encounter Profile Questionnaire Question 3 from baseline to after treatment, with an effect comparable to that of the phosphodiesterase inhibitor tadalafil given at the dose of 5 mg [208]. The improving effect of l-arginine on erectile dysfunction in men revealed by this and other studies is in line with the animal studies reviewed before, which show that this amino acid is able to reverse the inhibitory effect of competitive NO synthase inhibitors such L-NAME and L-NMMA on penile erection and copulatory behavior not only when given systemically but also in the PVN and the medial preoptic area, although it induces only modest effects when given alone [38,41,85] (see Section 2.2.2 and Section 3.1). Conversely, classic clinically used NO donors have produced contrasting and even negative results in double-blind crossover trials when tested for the treatment of human erectile dysfunction [133]. As recalled above, this may be due to the fact that NO is a potent vasorelaxing modulator present in all vascular tissues, and this may cause severe complications (e.g., marked hypotension). Whether this picture is going to change with the use of the new NO donors under development cited above (Section 2.2.1 and Section 2.2.2) [138,139,140] is still unknown.

## 6. Concluding Remarks

The studies reviewed suggest a key role of NO in the PVN for the control of erectile function and of both the PVN and the medial preoptic area in male rat sexual behavior. In the PVN NO is synthesized by NO synthase located in the cell bodies of oxytocinergic neurons projecting to extra-hypothalamic brain areas and mediating this sexual response. Accordingly, NO synthase is activated by agents supposed to induce penile erection by acting on these neurons in the PVN by increasing Ca^2+^ influx in their cell bodies (Figure 2). In fact, the activation of NO synthase is necessary for the induction of the sexual response induced by dopamine agonists, oxytocin, NMDA, hexarelin peptide analogues and VGF-derived peptides, since penile erection does not occur when the enzyme has been previously inhibited, for instance by NO synthase inhibitors injected in the PVN. Once formed, NO in the PVN activates yet unidentified intracellular targets, apparently different from guanylate cyclase (i.e., ADP-ribosyl-transferases and other iron-containing enzymes [4,12,26]), which in turn lead to the activation of oxytocinergic neurons mediating the appearance of the sexual response by releasing oxytocin in sites distant from the PVN, i.e., the ventral tegmental area, hippocampus, amygdala, ventral medulla and the spinal cord [40,53,54,56,57]. Interestingly, NO seems to act as an intracellular rather than an intercellular messenger, since penile erection is not prevented by NO scavenging from the PVN extracellular space with hemoglobin [96,97,103,104,143]. The pro-erectile effect of NO in the PVN is further confirmed by the ability of several NO donors to induce penile erection when injected into this nucleus. Additionally, in this case, a NO-cGMP pathway seems not to be involved, as the erectile response by NO donors is not abolished by NO scavenging or guanylate cyclase inhibition. Despite the importance of a normal function of NO in the PVN in the expression of penile erection, there are several pieces of evidence that NO is also involved in the control of this sexual response in sites different from the PVN. Among these are the ventral tegmental area, the ventral subiculum of the hippocampus, the posteromedial cortical nucleus of the amygdala, the bed nucleus of the stria terminalis (Figure 4) and even the ventral medulla and the spinal cord. In the majority of these areas NO is involved in the induction of penile erection induced by oxytocin. Accordingly, oxytocin injected into these areas induces penile erection which occurs with a concomitant increase in NO production measured by the increase in NO_2_^−^ and NO_3_^−^ found in the dialysate collected from these brain areas. At variance from the PVN, in the ventral tegmental area oxytocin induces penile erection by activating mesolimbic/mesocortical dopaminergic neurons co-expressing both NO synthase and guanylate cyclase, thus activating a NO-cGMP pathway in the same neurons in which NO is formed and acts as an intracellular messenger. Accordingly, (i) the NO synthase inhibitor S-methyl-thio-citrulline injected in this area before oxytocin antagonized both oxytocin-induced penile erection and the concomitant increase in NO production that occurs in the ventral tegmental area dialysate, and (ii) the active cGMP analogue 8-Bromo-cGMP induces penile erection, while the inhibitor of guanylate cyclase ODQ prevents oxytocin-induced penile erection without antagonizing the increase in NO production that occurs in the ventral tegmental area, in line with the hypothesis that in dopaminergic cell bodies cGMP acts downstream to NO [47,49,50,51]. As for the increased NO production that occurs in the ventral subiculum of the hippocampus or in the posteromedial cortical nucleus of the amygdala that leads to penile erection after the oxytocin injection in these two brain areas, the available data support the hypothesis that NO is produced in the cell bodies of glutamatergic neurons from which it is released in the extracellular space and acts as a intercellular messenger to activate glutamatergic neurons projecting directly or indirectly to the ventral tegmental area. Accordingly, oxytocin-induced penile erection and the concomitant increase in NO production that occur in the ventral subiculum are abolished not only by the prior blockade of oxytocinergic receptors and NO synthase inhibition but also by NO scavenging with hemoglobin. Further experiments are necessary to verify if a similar mechanism also takes place in the posteromedial cortical nucleus of the amygdala and/or in the bed nucleus of the stria terminalis. Two other sites in which NO may be involved in the control of erectile function are the ventral medulla and the spinal cord. Accordingly, NO synthase has been identified in these structures [52,127]. Since penile erection induced by 5HT_1C_ agonists is prevented dose dependently by NO synthase inhibitors given i.c.v. but not in the PVN [83], this raises the possibility that NO may be involved in the expression of penile erection induced by these compounds in these two areas [80]. Likewise, it is possible that NO synthase inhibitors prevent ACTH-induced penile erection [41] by acting in a yet undiscovered brain area.

In the medial preoptic area, NO is formed in NO synthase-containing neurons/cells that are activated to facilitate the release of dopamine by the nerve endings of glutamatergic neurons originating in the amygdala and the bed nucleus of the stria terminalis, which are stimulated by sexual (mainly olfactory, i.e., pheromones) stimuli [178,179,183,184,185]. Glutamic acid activates dopamine release by acting on NMDA receptors coupled to voltage-dependent Ca^2+^ channels in still unknown medial preoptic neurons/cells. The increased Ca^2+^ influx activates NO synthase to increase NO production. Once released, NO in turn travels as a retrograde messenger to dopaminergic synapses and possibly also to the glutamatergic synapses, where it activates guanylate cyclase to increase cGMP levels to facilitate dopamine release to improve copulation and sustain glutamic acid release to maintain dopamine release (Figure 5) [39,178,179,180,181,182,183,184,185]. Accordingly, in male rats (i) NO synthase inhibitors, when injected into the medial preoptic area, abolish both dopamine and glutamic acid release that occur in the medial preoptic area during copulation and impair sexual behavior; (ii) 8-Bromo-cGMP facilitates dopamine release and copulation, while ODQ inhibits glutamic acid and dopamine release and copulation as well (Table 10) [181,182,183]. NO facilitates sexual behavior in male rats by acting not only in the medial preoptic area, but also in the PVN. Accordingly, (i) NO production increases in the PVN when penile erection occurs in physiological contexts; that is, when male rats show non-contact erections in the presence of an inaccessible sexually receptive female or when it occurs during copulation, and (ii) inhibition of NO synthase in the PVN prevents non-contact erections and impairs copulation. However, at variance from the medial preoptic area, in the PVN NO facilitates sexual behavior by a yet undiscovered mechanism not involving cGMP [74].

**Figure 6:**
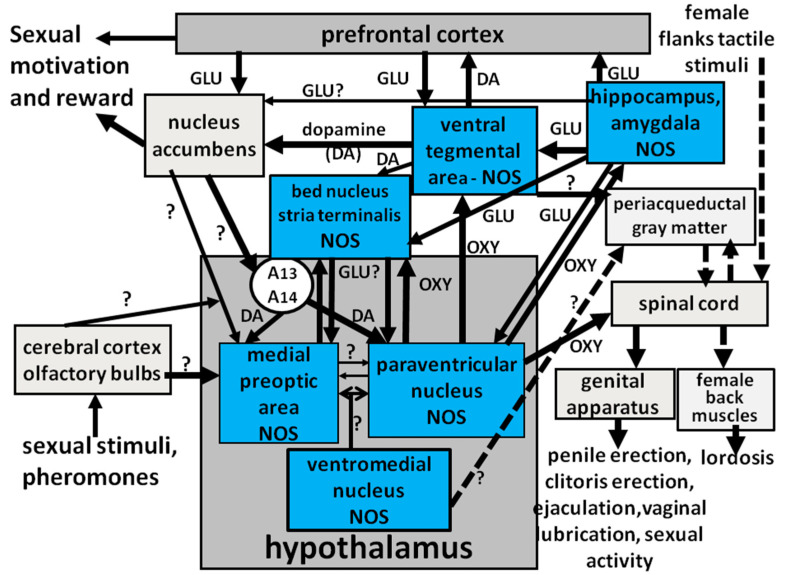
Nitric oxide produced by NO synthase (NOS) participates in a complex neural circuit that controls sexual motivation, reward and performance at central level together with other neurotransmitters and neuropeptides. This circuit interconnects the hypothalamus and its nuclei (paraventricular and ventromedial) with limbic areas (ventral tegmental area, hippocampus, amygdala, bed nucleus of the stria terminalis) and with the ventral medulla and spinal cord. Among neural pathways involved are (i) PVN oxytocinergic neurons that send their projections to the ventral medulla and spinal cord, and to the ventral tegmental area, hippocampus, amygdala and bed nucleus of the stria terminalis; (ii) mesolimbic/mesocortical dopaminergic neurons originating in the ventral tegmental area that send projections to the nucleus accumbens, medial prefrontal cortex, bed nucleus of the stria terminalis, hippocampus and amygdala; (iii) glutamatergic neurons that interconnect the hippocampus and the amygdala with the ventral tegmental area, prefrontal cortex, bed nucleus of the stria terminalis, medial preoptic area and PVN; (iv) neurons that project from the ventromedial nucleus of the hypothalamus to the periaqueductal gray matter and from here to the spinal cord (dashed lines), which control lordosis in estrous females and whose activity is modulated by different brain areas (medial preoptic area, ventral tegmental area, lateral septum). When this circuit is activated by physiological stimuli (i.e., pheromones released by sexually receptive females in males or by sexually potent males in females and/or other sexual stimuli) or by drugs or peptides given in one of the areas of the circuit, NO becomes involved at different sites, i.e., in the paraventricular and ventromedial nuclei and medial preoptic area, which are involved more in sexual performance (penile erection and copulation in males, lordosis in females), and the ventral tegmental area, hippocampus, amygdala and bed nucleus of the stria terminalis, which act on mesolimbic/mesocortical dopaminergic neurons that control sexual motivation, arousal and reward (light blue boxes indicate the brain areas of the circuit containing NO synthase). Thus, changes in NO activity in these brain areas participate in the modulation of both sexual motivation/arousal/reward and sexual performance (erectile function/lordosis/copulation). (?) = unknown neurotransmitters/neuropeptides. Details are found in [4,16,17,26,47,49,53,54,55,56,57,64,65,66,67,68,69,70,96,97,98,99,104,143,144,151,152,160,174,179,183,184,185,190,192,193,194] and references therein.

The results reviewed in this work show also that NO plays a key role in the control of erectile function at central level by acting as a common mediator of several neurotransmitters and neuropeptides that control this sexual function. This occurs at level of the PVN, which is considered a sort of integration center between the central and the peripheral autonomous nervous systems [209], and at the level of the limbic system, mainly in the ventral tegmental area, which is, among others, strictly connected with the hippocampus, the amygdala, the bed nucleus of the stria terminalis and the medial preoptic area [53,54,56,57]. Most importantly, the activation of mesocorticolimbic dopaminergic neurons by oxytocin injected in the ventral tegmental area leads to the release of dopamine in the nucleus accumbens [47,48,49]. This in turn causes an increase in dopamine release from incertohypothalamic neurons that impinge on oxytocinergic neurons in the PVN sending projections back to the ventral tegmental area, the hippocampus, the amygdala, the bed nucleus of the stria terminalis, the medial preoptic area and the spinal cord leading to penile erection [57,144]. Together these findings support the hypothesis that NO plays a main role at several levels in a complex neural circuit that interconnects these brain areas and contains the neuronal systems responsible of the anticipatory phase (mediating sexual motivation and reward) with those responsible of the consummatory phase (mediating copulation and sexual performance) of sexual behavior (Figure 6) [53,54,55]. In view of the existence of a similar distribution of central oxytocinergic neurons between male and female rats, it is feasible to assume that the neural circuit described above is also involved in female sexual behavior, although with some differences between male and female rats. The most important difference regards the role of the medial preoptic area, which exerts inhibitory control on female rat sexual behavior rather than facilitatory as extensively reported for male rat sexual behavior (see Section 3). In fact, at variance from the ventromedial nucleus of the hypothalamus, the activation of the medial preoptic area in sexually receptive (estrogen-primed) female rats inhibits rather than facilitates the lordotic reflex, while the opposite is seen after lesions of this brain area [191,210]. The medial preoptic area inhibitory effect on lordosis is apparently mediated by the activation of neurons projecting to the ventral tegmental area and from here to the periaqueductal gray matter [210], which receives the sensory inputs produced by tactile stimulation of the female flanks and/or perineal regions from the lumbosacral L1-S1 spinal tract to coordinate the striated muscles activity leading to the lordotic posture [191,193,194,195]. Irrespective of the different role the medial preoptic area may have in male and female rat sexual behavior, and of the fact that lordosis is mediated by neural pathways different from those that mediate erectile function and ejaculation in males and clitoris erection and vaginal lubrication in females [190], it is reasonable to assume that this neural circuit and its components, including NO, not only contribute to the consummatory aspects of sexual behavior (erectile function and copulation in males and lordosis in females), but also in the same time activate mesolimbic/mesocortical dopaminergic neurons providing a neural substrate for explaining the motivational and rewarding properties of sexual activity (Figure 6). In agreement with this possibility, the central administration of NO synthase inhibitors decreases the percentage of copulating male rats and impairs the indexes of sexual activity in sexually experienced and naive male rats [38,41,175]. More intriguing and relevant to the mechanisms controlling erectile function and sexual behavior considered in this review, the NO synthase messenger RNA expression is found to be in the PVN of sexually potent male rats about twice that of impotent male rats [41]. In view of the importance of the PVN in the expression of penile erection, it was suggested in 1995 that NO was deeply involved in the control of male sexual behavior and that the inhibitory effect of NO synthase inhibitors given centrally on male copulatory performance might be due to the inhibition of erectile function secondary to the inhibition of central oxytocinergic transmission at the PVN level [211]. However, since the activation of NO synthase facilitates erectile function and sexual behavior by acting not only in the PVN, but also in many other brain areas (i.e., the ventral tegmental area, ventral subiculum of the hippocampus, amygdala-posteromedial cortical nucleus, bed nucleus of the stria terminalis, medial preoptic area and spinal cord) (see above), it is evident that the role on NO in erectile function and sexual behavior is more diffused across the brain than was thought in the 1990s. Although difficult to translate to humans, the increased knowledge obtained in laboratory animals, mainly rodents, on the sexual role of NO at central level will contribute to finding new strategies for the therapy of sexual dysfunctions and other sexual disorders in humans.

## Figures and Tables

**Table 1:** Drug- and peptide-induced penile erection in male rats: effect of NO synthase inhibitors given systemically, intracerebroventricularly or in the PVN.

Penile Erection Induced by	L-NAME			Effect on Penile Erection L-NMMA			D-NMMA		
	Given			Given			Given		
	i.p.	i.c.v	PVN	i.p.	i.c.v.	PVN	i.p.	i.c.v.	PVN
Dopamine agonists	↓	↓	↓	↓	↓	↓	==	==	==
Oxytocin	↓	↓	↓	↓	↓	↓	==	==	==
NMDA	↓	↓	↓	↓	↓	↓	==	==	==
5-HT_1C_ agonists	↓	↓	==	↓	↓	==	==	==	==
ACTH 1-24	↓	↓	==	↓	↓	==	==	==	==
Hexarelin peptides	n.a.	n.a.	↓	n.a.	n.a.	↓	n.a.	n.a.	==
VGF peptides	n.a.	n.a.	↓	n.a.	n.a.	↓	n.a.	n.a.	==
SR 141716A	n.a.	n.a.	↓	n.a.	n.a.	↓	n.a.	n.a.	==

↓ = prevention; == = no effect; n.a. = not available; i.p. = intraperitoneally; i.c.v. = intracerebroventricularly; PVN = into the PVN. Dopamine agonists = dopamine receptor agonists of the D2 receptor family (D_2_ and D_4_ receptor subtype); L-NAME = N^G^-nitro-l-arginine methyl ester; L-NMMA = N^G^-monomethyl-l-arginine; d-NMMA = N^G^-monomethyl-d-arginine. Dopamine agonists were given systemically or into the PVN, oxytocin i.c.v. or in the PVN, N-methyl-D-aspartic acid in the PVN, 5-HT_1C_ agonists systemically or into the PVN, ACTH 1-24 i.c.v., hexarelin analogues, VGF-derived peptides and SR 141716A into the PVN. Details are found in [36,37,38,41,43,79,80,81,82,83,84,86,90,91,93,96,97,98,99,100,101].

**Table 2:** Effect of L- and d-arginine and NO donors given i.c.v. or into the PVN on penile erection in male rats.

	Penile Erection	
NO Donor Given	i.c.v.	Into the PVN
l-arginine	==	↑
d-arginine	==	==
Nitroglycerin	↑	↑
Sodium nitroprusside	==	↑
Isoamyl nitrite	↑	n.a.
Hydroxylamine	↑	n.a.
S-nitroso-D-acetyl-penicillamine	n.a.	n.a.

↑ = increase; == = no effect; n.a. = not available. Details are found in [37,85,86,103,141].

**Table 3:** Dopamine agonists (of the D_2_ and D_4_ receptor subtype), oxytocin, NMDA, hexarelin analogue peptides, VGF-related peptides injected into the PVN of male rats induce penile erection and increase NO production in the PVN: effect of NO synthase inhibitors (L-NAME or SMTC) and/or drug/peptide receptor antagonists given in the PVN or i.c.v. when indicated.

PVN Pretreatment	PVN Treatment	NO Production	Penile Erection
Vehicle/Drug/Peptide	Drug/Peptide		
L-NAME/SMTC	DA agonists	↓	↓
Haloperidol	DA agonists	↓	↓
SCH 23390	DA agonists	==	==
Oxy-Ant	apomorphine	==	==
OXY-Ant i.c.v.	apomorphine	==	↓
(+)MK-801	apomorphine	==	==
L-NAME/SMTC	oxytocin	↓	↓
Haloperidol	oxytocin	==	==
Oxy-Ant	oxytocin	↓	↓
Oxy-Ant i.c.v.	oxytocin	↓	↓
(+)MK-801	oxytocin	==	==
L-NAME/SMTC	NMDA	↓	↓
Haloperidol	NMDA	==	==
Oxy-Ant	NMDA	==	==
Oxy-Ant i.c.v.	NMDA	==	↓
(+)MK-801	NMDA	↓	↓
L-NAME	VGF 588-617	↓	↓
Cis-flupenthixol	VGF 588-617	n.a.	==
Oxy-Ant	VGF 588-617	==	==
Oxy-Ant i.c.v.	VGF 588-617	==	↓
(+)MK-801	VGF 588-617	n.a.	==
L-NAME	EP 80661	↓	↓
Cys-flupenthixol	EP 80661	n.a.	==
Oxy-Ant	EP 80661	==	==
Oxy-Ant i.c.v.	EP 80661	==	↓
(+)MK-801	EP 80661	n.a.	==

↓ = prevention; == = no effect; n.a. = not available; DA agonists = dopamine agonists of the D2 receptor family (D2 and D4 receptor subtype); haloperidol, cys-flupenthixol and SCH23390 are dopamine receptor antagonists of the D2, D1/D2 and D1 receptors, respectively; L-NAME = N^G^-nitro-l-arginine methyl ester; SMTC = S-methyl-thio-citrulline; NMDA = N-methyl-D-aspartic acid, (+)MK-801 is a NMDA receptor antagonist; Oxy-Ant is d(CH_2_)_5_Tyr(Me)-Orn^8^ vasotocin, an oxytocin receptor antagonist; EP 80661 is an hexarelin peptide analogue, VGF 588-671 is a VGF derived peptide. Details are found in [36,37,79,80,81,82,83,88,93,96,97,98,99,102,104,143,149,150].

**Table 4:** NO production increases in the PVN of male rats during non-contact erections and copulation: reversal by the NO synthase inhibitor L-NAME, GABA agonists and the opiate morphine but not by methylene blue, hemoglobin, or d(CH_2_)_5_Tyr(Me)-Orn^8^ vasotocin (Oxy-Ant) injected into the PVN, in spite of the ability of the latter to abolish non-contact erections and impair copulation when given i.c.v.

PVN Treatment	NO Production	Non-Contact Erections	Copulation
L-NAME	↓	↓	↓
Muscimol	↓	↓	↓
Baclofen	==	==	==
Morphine	↓	↓	↓
U-69,593	==	==	==
Methylene Blue	==	==	==
Hemoglobin	==	==	==
Oxy-Ant	==	==	==
Oxy-Ant i.c.v.	==	↓	↓

↓ = prevention; == = no effect; n.a. = not available; L-NAME = N^G^-nitro-l-arginine methyl ester; muscimol is a GABA_A_ receptor agonist, baclofen a GABA_B_ receptor agonist, morphine a µ-opioid receptor agonist, U-69,593 a k-opioid receptor agonist, methylene blue a guanylate cyclase inhibitor, hemoglobin a NO scavenger and d(CH_2_)_5_Tyr(Me)-Orn^8^ vasotocin (Oxy-Ant) an oxytocin receptor antagonist. Details are found in [77,104,105,107,114,115,116].

**Table 5:** Dopamine agonists (of the D_2_ and D_4_ receptor subtype), oxytocin, NMDA, hexarelin analogue peptides, VGF-related peptides injected into the PVN induce penile erection and increase NO production in the PVN of male rats: reversal by GABA agonists and the opiate morphine injected into the PVN.

PVN Pretreatment	PVN Treatment	NO Production	Penile Erection
Vehicle/Drug/Peptide	Drug/Peptide		
Muscimol	DA agonists	↓	↓
Baclofen	DA agonists	==	==
Morphine	DA agonists	↓	↓
U-69,593	DA agonists	==	==
Muscimol	oxytocin	↓	↓
Baclofen	oxytocin	==	==
Morphine	oxytocin	↓	↓
U-69,593	oxytocin	==	==
Muscimol	NMDA	↓	↓
Baclofen	NMDA	==	==
Morphine	NMDA	↓	↓
U-69,593	NMDA	n.a.	==
Muscimol	VGF 588-617	↓	↓
Baclofen	VGF 588-617	n.a.	==
Morphine	VGF 588-617	n.a.	↓
U-69,593	VGF 588-617	n.a.	==
Muscimol	EP 80661	↓	↓
Baclofen	EP 80661	n.a.	==
Morphine	EP 80661	↓	↓
U-69,593	EP 80661	n.a.	==

↓ = prevention; == = no effect; n.a. = not available. DA agonists are dopamine receptor agonists of the D2 receptor family (D_2_ and D_4_ receptor subtype); NMDA N-methyl-D-aspartic acid; muscimol a GABA_A_ receptor agonist, baclofen a GABA_B_ receptor agonist, morphine a µ-opioid receptor agonist, U-69,593 a k-opioid receptor agonist. EP 80661 a hexarelin peptide analogue, VGF 588-617 a VGF derived peptide. Details are found in [106,108,109,110,111,112,116].

**Table 6:** NO facilitates erectile function and sexual behavior in rats by acting in different brain areas and with different mechanisms: a brief summary.

Brain Area	NO Synthesis Site	Mechanism of Action
PVN	Cell bodies of oxytocinergic neurons projecting to extrahypohalamic brain areas and spinal cord	Activation of central oxytocin neurotransmission apparently by acting as intracellular messenger and with a mechanism that does not involve guanylate cyclase yet to be identified.
Medial preoptic area	Medial preoptic cells yet to be identified	Activation of dopamine release by insert hypothalamic dopaminergic neurons by acting as a retrograde messenger with a mechanism that involves the activation of guanylate cyclase.
Ventromedial nucleus of the hypothalamus	Neurons yet to be identified	Activation of the lordosis response by a still unknown mechanism.
Ventral tegmental area	Cell bodies of mesolimbic/mesocortical dopaminergic neurons	Activation of mesolimbic dopaminergic neurons projecting to the nucleus accumbens and medial prefrontal cortex by acting as intracellular messenger with a mechanism that involves the activation of guanylate cyclase.
Ventral subiculum of the hippocampus	Glutamatergic neurons of the subiculum	Activation of glutamatergic neurons projecting directly or indirectly (through the medial prefrontal cortex) to the ventral tegmental area in order to activate mesolimbic dopaminergic neurons by acting as intercellular messenger with a mechanism involving guanylate cyclase.
Posteromedial cortical nucleus of the amygdala	Glutamatergic neurons of the amygdalar nucleus	Activation of glutamatergic neurons projecting directly or indirectly (through the medial prefrontal cortex) to the ventral tegmental area in order to activate mesolimbic dopaminergic neurons by acting as intercellular messenger, possibly with a mechanism involving guanylate cyclase.
Bed nucleus of the stria terminalis	Glutamatergic neurons of the bed nucleus	Activation of glutamatergic neurons projecting to the PVN and other extrahypothalamic brain areas mediating erectile function. It is still unknown if NO acts as intercellular or intracellular messenger and if guanylate cyclase is involved or not.
Ventral medulla (nucleus paragigantocellularis	5-HT cell bodies of neurons projecting to the spinal cord (L2-S2)?	Activation of 5HT neurons projecting from the ventral medulla to the spinal tract L2-S2, with a still-unknown mechanism?
Spinal cord (L2-S2)	Spinal neurons of the L2-S2 spinal tract	Activation of spinal neurons projecting to the pelvic plexuses and pudendal nerves that reach the penis to control cavernous corpora relaxation and the muscles at the basis of the penis involved in penile reflexes and reflex erection.

**Table 7:** Effect of L- and d-arginine and NO donors given i.c.v. or into the PVN or after pretreatment with the NO synthase inhibitor L-NAME or the oxytocin receptor antagonist d(CH_2_)_5_Tyr(Me)-Orn^8^-vasotocin (Oxy Ant) given i.c.v or into the PVN on penile erection in male rats.

		Penile Erection		
i.c.v. Pretreatment	PVN Pretreatment	NO Donor Given	i.c.v	PVN
Vehicle	Vehicle			
Drug	Drug			
Peptide	Peptide			
Saline		d-arginine	==	
	Saline	d-arginine		==
Saline		l-arginine	==	
	Saline	l-arginine		↑
Oxy-Ant		l-arginine	↓	
	Oxy-Ant	l-arginine		↑
L-NAME		l-arginine	↓	
	L-NAME	l-arginine		↓
Vehicle		Nitroglycerin	↑	
	Vehicle	Nitroglycerin		↑
Oxy-Ant		Nitroglycerin	↓	
	Oxy-Ant	Nitroglycerin		↑
L-NAME		Nitroglycerin	↑	
	L-NAME	Nitroglycerin		↑
	vehicle	SNP		↑
Oxy-Ant		SNP		↓
	Oxy-Ant	SNP		↑
L-NAME		SNP		↑
	L-NAME	SNP		↑
Vehicle		Isoamyl nitrite	↑	
Oxy-Ant		Isoamyl nitrite	↓	
L-NAME		Isoamyl nitrite	↑	
Vehicle		Hydroxylamine	↑	
Oxy-Ant		Hydroxylamine	↓	
L-NAME		Hydroxylamine	↑	

↓ = prevention; ↑ increase; == = no effect; n.a. = not available. SNP = sodium nitroprusside. Details are found in [37,85,103,136].

**Table 8:** PVN drug-, peptide- and NO donor-induced penile erection and PVN NO production in male rats: effects of guanylate cyclase (GC) inhibitors (methylene blue or LY 83583) and the NO scavenger hemoglobin given i.c.v. or into the PVN.

Drug, Peptide, NO Donor into the PVN	Penile Erection				NO Production			
	GC Inhibitors		Hemoglobin		GC Inhibitors		Hemoglobin	
	Given				Given			
	i.c.v.	PVN	i.c.v.	PVN	i.c.v.	PVN	i.c.v.	PVN
DA agonists	↓	==	==	==	==	==	↓	↓
Oxytocin	↓	==	==	==	==	==	↓	↓
NMDA	↓	==	==	==	==	==	↓	↓
EP 80661	↓	==	==	==	n.a.	n.a.	↓	↓
VGF 588-617	↓	==	==	==	n.a.	n.a.	↓	↓
5-HT_1C_ agonists	↓	==	==	==	n.a.	n.a.	↓	↓
NO donors	↓	==	==	==	n.a.	n.a.	↓	↓

↓ = prevention; == = no effect; n.a. = not available. GC inhibitors for methylene blue or LY 83586, DA agonists for dopamine receptor agonists of the D2 family (D_2_ and D_4_ receptor subtype); NMDA for N-methyl-D-aspartic acid; EP 80661 is a hexarelin peptide and VGF 678-617 is a VGF peptide. Details are found in [82,83,85,86,90,91,92,93,96,97,103,104,143].

**Table 9:** Oxytocin-induced penile erection and NO production increase in the ventral tegmental area and the ventral subiculum of male rats: effect of the NO synthase inhibitor L-NAME, the oxytocin receptor antagonist d(CH_2_)_5_Tyr(Me)-Orn^8^-vasotocin (Oxy-Ant), the guanylate cyclase inhibitor ODQ and the NO scavenger hemoglobin given in the ventral tegmental area and in the ventral subiculum, respectively.

	Oxytocin Injected in the			
Pretreatment	Ventral Tegmental Area		Ventral Subiculum	
	Penile Erection	NO Production	Penile Erection	NO Production
L-NAME	↓	↓	↓	↓
Oxy-Ant	↓	↓	↓	↓
ODQ	↓	==	n.a.	n.a.
Haemoglobin	==	↓	↓	↓

↓ = prevention; == = no effect; n.a. = not available. Details are found in [47,48,49,50,51,160].

**Table 10:** NO facilitates copulatory behavior by increasing dopamine release in the medial preoptic area of male rats engaged in copulation with a sexually receptive female: effect of NO synthase inhibitors (L-NAME and L-NMMA), NO donors (SNP), guanylate cyclase inhibitors (ODQ) and 8-Bromo-cGMP injected into the medial preoptic area on extracellular dopamine and copulation.

MPOA Treatment	Dopamine	Copulation
l-arginine	↑	↑
L-NAME	↓	↓
D-NAME	↑	↑
L-NAME+l-Arginine	↓	↓
SNP	↑	↑
ODQ	↓	↓
ODQ+SNP	↓	↓
8-Bromo-cGMP	↑	↑
L-NMMA+8-Bromo-cGMP	↑	↑

↑ = increase; ↓ = prevention; n.a. = not available. L-NAME is N^G^-nitro-l-arginine methyl ester, L-NMMA is N^G^-monomethyl-l-arginine; SNP is sodium nitroprusside and ODQ a guanylate cyclase inhibitor. Details are found in [38,39,44,45,175,176,177,178,179,180,181,182,183,184,185].
